# Recent Advances in Intramolecular C─N Bond Formation for Pyrrolidine Synthesis

**DOI:** 10.1002/open.70182

**Published:** 2026-05-19

**Authors:** Rasma Kroņkalne, Māris Turks

**Affiliations:** ^1^ Institute of Chemistry and Chemical Technology Faculty of Natural Sciences and Technology Riga Technical University Riga Latvia

**Keywords:** C─H activation, C─N bond formation, heterocyclization, intramolecular amination, pyrrolidines

## Abstract

Pyrrolidines constitute a privileged class of nitrogen heterocycles that are ubiquitous in pharmaceuticals, bioactive molecules, and functional materials, thereby continuing to stimulate the development of efficient and versatile synthetic strategies. Among these, intramolecular C─N bond formation represents one of the most fundamental and broadly applicable approaches for the construction of pyrrolidine frameworks. Despite its central importance, a systematic overview focusing specifically on this ring‐closing paradigm has been lacking. This review summarizes key advances reported between 2013 and 2025 in the synthesis of pyrrolidines via intramolecular C─N bond formation, focusing specifically on examples featuring a polar ring‐closure step N: → C(+/δ^+^). The discussion is organized according to the ring‐formation mechanism, encompassing transition‐metal‐catalyzed and metal‐free cyclization of alkenyl and alkynyl amines, tandem annulation processes, intramolecular nucleophilic additions to carbonyl compounds, nucleophilic substitution reactions, and regioselective C(sp^3^)‐H activation of aliphatic amines, including Hofmann‐Löffler‐Freytag‐type transformations. By highlighting mechanistic features, substrate scope, and synthetic utility, this review aims to provide a comprehensive and practical reference for the design of new pyrrolidine syntheses based on intramolecular C─N bond formation.

## Graphical abstract

This review highlights synthetic advances (2013–2025) in intramolecular C─N bond formation for constructing the pharmaceutically relevant pyrrolidine core. The review is organized into five methodological clusters: 1) intramolecular alkene/alkyne amination; 2) tandem annulations, which involve C─N bond formation as the key step; 3) nucleophilic additions to carbonyl moieties; 4) intramolecular nucleophilic substitution; and 5) C(sp^3^)‐H activation in aliphatic amines.

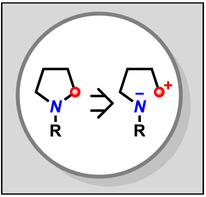

## 1 Introduction

The pyrrolidine scaffold is a privileged structural motif in medicinal chemistry and ranks among the most frequently encountered frameworks in small‐molecule drugs [1, 2] and bioactive compounds [3, 4, 5]. Over the past decade, pyrrolidines have remained prominent targets in synthetic chemistry, motivating the development of a wide array of methodologies for the preparation of structurally diverse derivatives. Several recent target‐oriented reviews have highlighted the synthesis of pyrrolidine‐based organocatalysts [6], pharmaceutical agents and their precursors [7], spiro‐pyrrolidines [8, 9], and other pyrrolidine scaffolds bearing specific substitution patterns [10, 11, 12, 13, 14, 15]. Complementary methodology‐focused reviews have summarized advances in photochemical approaches [16, 17], Lewis‐acid‐catalyzed cyclizations of propargyl and homopropargyl amines [18], enantioselective silver‐catalyzed reactions [19], copper‐catalyzed alkyne aminations [20], glycine‐based [3 + 2] cycloadditions for the synthesis of polycyclic architectures [21], multicomponent reactions [22], palladium‐catalyzed annulations [23], and oxidative addition of sulfonamides to allylic substrates [24].

In this review, we aim to showcase the diversity of modern synthetic strategies for constructing the pyrrolidine framework via intramolecular C─N bond formation. This transformation, typically proceeding through a cationic mechanism, occurs in polar systems that combine a nucleophilic nitrogen center with an electrophilic carbon species. Representative substrates include alkenyl and alkynyl amines, γ‐amino carbonyl compounds, amino alcohols, and sulfinimines bearing suitable leaving groups. Furthermore, through C─H activation pathways, even aliphatic amines can be induced to cyclize via intramolecular C─N bond formation. Despite the fundamental importance of this ring‐closing strategy in contemporary pyrrolidine synthesis, a comprehensive review focusing specifically on intramolecular C─N bond formation as the defining step in pyrrolidine construction was lacking.

**SCHEME 1:**
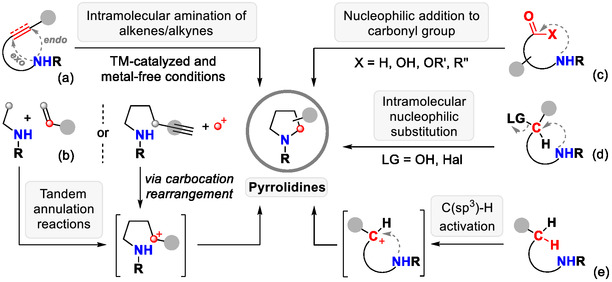
Overview on intramolecular C─N bond formation reactions for pyrrolidine synthesis.

Herein, we summarize key developments reported between 2013 and 2025 in the field of intramolecular C─N bond formation leading to pyrrolidine frameworks. The review is organized into five thematic sections: (a) transition‐metal‐catalyzed and metal‐free activation of electrophilic carbon centers (C^+^/C^δ+^) in alkenyl and alkynyl amines; (b) tandem annulation reactions featuring C─N bond formation as the pivotal step; (c) intramolecular nucleophilic additions to carbonyl compounds; (d) intramolecular nucleophilic substitution reactions; and (e) regioselective C(sp^3^)–H activation of aliphatic amines, including Hofmann–Löffler–Freytag‐type transformations (Scheme 1).

## 2 Intramolecular Amination of Double/Triple Bonds

Intramolecular amination of unsaturated C─C bonds might be the most extensively studied heterocyclization approach in the past decade, leading to pyrrolidines bearing diverse substitution patterns. For simplicity, we categorized the relevant examples by their cyclization mechanism according to Baldwin's rules [25]. In this section, we discuss both transition‐metal (TM)‐catalyzed and metal‐free activation conditions to enable intramolecular amination, including examples for asymmetric synthesis.

### 2.1 5‐*Exo*‐*Trig*‐Cyclization

**SCHEME 2:**
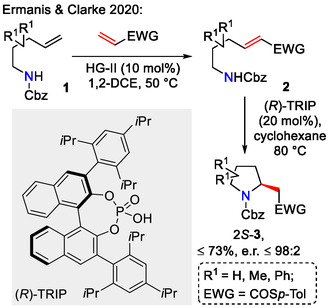
Asymmetric synthesis of pyrrolidines by chiral phosphoric acid‐catalyzed *aza*‐Michael reaction.

Olefin C=C bond polarization by the introduction of an electron‐withdrawing substituent (EWG) enables intramolecular amination in *aza*‐Michael fashion. While such transformations are common in their racemic version, the number of asymmetric examples is still limited [26]. Asymmetric *aza*‐Michael cyclizations have found applicability in the synthesis of natural products and biologically active heterocycles [27, 28, 29, 30]. One of such asymmetric examples, incorporating cyclization via the *5‐exo‐trig‐*mechanistic pathway, was demonstrated by Ermanis and Clarke, where cross‐metathesis between amine‐containing alkenes **1** and thioacrylate using the Hoveyda‐Grubbs second‐generation catalyst (HG‐II) provided EWG‐activated alkenes **2**, and the latter underwent intramolecular *aza*‐Michael cyclization to give pyrrolidine derivatives **3** (Scheme 2). The cyclization step was catalyzed by (*R*)‐TRIP, a chiral phosphoric acid, to obtain 2*S*‐pyrrolidines [26]. The opposite stereoselectivity was achieved with (*R*)‐TiPSY as the chiral acid catalyst, yielding 2*R*‐substituted pyrrolidines **3** [31].

**SCHEME 3:**
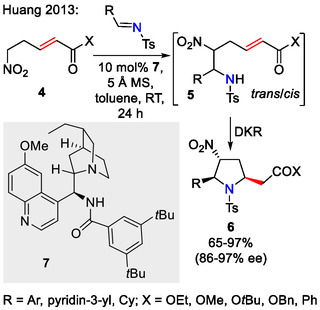
A DKR‐driven approach towards asymmetric synthesis of pyrrolidines.

The Huang group used a reversible *aza*‐Henry reaction, followed by dynamic kinetic resolution (DKR)‐driven *aza*‐Michael cyclization to obtain pyrrolidines **6** with up to three stereogenic centers (Scheme 3). Ee's up to 97% could be achieved by using Cinchona alkaloid‐derived amine/amide organocatalyst **7** [32].

**SCHEME 4:**
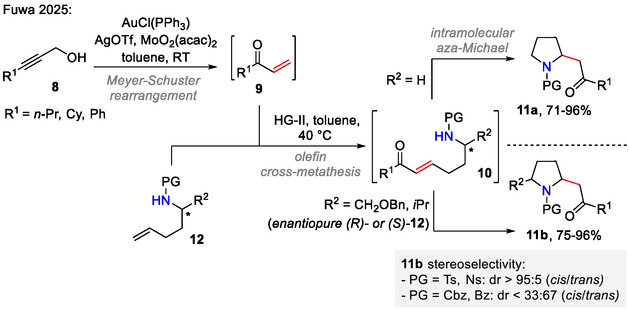
Tandem synthesis of 2‐carbonylmethyl pyrrolidines **11a** and their 2,5‐substituted analogs **11b**.

More recently, Fuwa and coworkers reported a tandem synthesis of 2‐carbonylmethyl pyrrolidines **11a** from propargyl alcohols **8** in a three‐step, one‐pot process (Scheme 4). First, the propargyl alcohol **8** was converted to vinyl ketone **9** by a Au/Mo‐catalyzed Meyer–Schuster reaction. Next, cross metathesis with amino olefin **12** using **HG‐II** catalyst afforded an α,β‐unsaturated ketone, which further participated in an intramolecular *aza*‐Michael addition to afford pyrrolidine **11a**. In some cases, where the *N*‐protected amino group was less nucleophilic (PG = Ns, Bz; *R* ^2^ ≠  H), catalytic amounts of Bi(OTf)_3_ were added to promote the cyclization step. In the absence of an additional Lewis acid, the cationic (Ph_3_P)Au^+^ catalyst promoted the *aza*‐Michael addition step. α,β‐Unsaturated ketones, derived from enantiopure amino olefins (*R*)‐ or (*S*)‐**12** (*R* ^2^ ≠ H), cyclized to afford 2,5‐disubstituted pyrrolidines **11b**, where *cis/trans* selectivity was dependent on the amine protecting group. Cbz‐ or Bz‐protected amines gave a *cis/trans* isomer mixture with slight preference for the *trans*‐pyrrolidine **11b** (dr < 33:67 *cis*/*trans*). Sulfonamides (PG = Ts, Ns), on the other hand, displayed excellent selectivity for *cis*‐product **11b** formation (dr > 95:5 *cis*/*trans*) [33].

As demonstrated by previous examples [26, 31, 32, 33], the *aza*‐Michael cyclization approach requires multistep synthesis of the α,β‐unsaturated ester/thioester/ketone intermediate, bearing an intrinsic amine moiety. The nucleophilic attack, however, occurs under relatively mild conditions, which is attributed to activation by electron withdrawing olefin substituents. Intramolecular amination of electron‐rich (unactivated) alkenes, however, is more challenging and often requires activation by transition metal catalysts [34]. Another possibility for electron‐rich olefin hydroamination is to employ photoredox catalysis, as demonstrated by Knowles group. They used aminium radical cation intermediates for the C─N bond construction [35].

**SCHEME 5:**
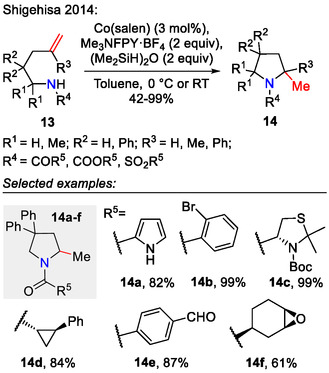
Pyrrolidine synthesis by Co‐catalyzed amination of alkene **13**.

The application of photoredox techniques for C─N bond formation in related systems was highlighted in recently published reviews [36, 37, 38]. Our focus being the C─N bond formation strategies proceeding via a cationic mechanism, we wanted to explore in more depth some of the transition metal‐catalyzed approaches. In one such example, Shigehisa et al. transformed unactivated alkenylamines **13** into 2‐methylpyrrolidines **14** by using the Co(salen) catalyst in combination with *N*‐fluoro‐2,4,6‐trimethylpyridinium tetrafluoroborate and disiloxane (Scheme 5). Exceptional functional group tolerance was observed under these conditions, allowing diverse *N*‐functionalization possibilities [39].

**SCHEME 6:**
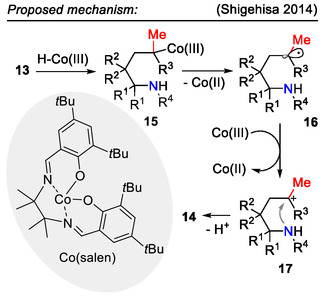
Proposed mechanism for Co‐catalyzed amination of alkene **13**.

In the proposed reaction mechanism (Scheme 6), the *N*‐fluoro‐2,4,6‐trimethylpyridinium salt fluorinated the Co(II) catalyst to form the F‐Co(III) intermediate, which was hydrosilylated to afford the active catalytic H‐Co(III) species. Cobalt(III) hydride was then added to the olefin **13** according to Markovnikov's rule. Homolytic cleavage of the C‐Co(III) bond afforded the radical intermediate **16**, which was oxidized to the carbocation **17** by the present Co(III) species. Lastly, the C─N bond was formed by nucleophilic attack of the tethered amide, affording the 2‐methylpyrrolidine product **14** [39].

**SCHEME 7:**
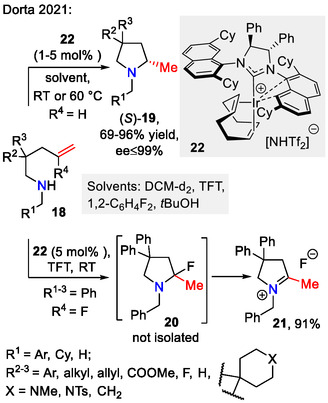
Asymmetric hydroamination of unactivated aminoalkenes **18**.

Asymmetric hydroamination of unactivated aminoalkenes **18** was achieved by Dorta and coworkers using a cationic iridium system **22** with the general formula [(NHC*)Ir(COD)][X] (Scheme 7). Noteworthy features of this method include high enantioselectivity, ensured by incorporation of a chiral monodentate NHC steering ligand, and high functional group tolerance, where excellent results could be achieved with both electron‐rich and poor benzylic substituents. *N*‐Alkyl substituents gave equally good results, although a slight decrease in enantioselectivity was observed with the *N*‐methyl substituted substrate **18** (*R* ^1^ = H, *R* ^2,3^ = Ph), indicating that a more sterically obstructing *N*‐substituent is favorable. Interestingly, switching to the similarly sized, but electronically distinct *N*‐phenyl substituent decreased reactivity and the resulting 2‐methylpyrrolidine **19** was no longer obtained in optically enriched form (*R* ^2,3^ = Ph, 92% yield, 9% ee), limiting this method to the *N*‐RCH_2_‐moiety containing substrates **18**. Fluorinated olefin **18** (*R* ^1–3^ = Ph, *R* ^4^ = F), when subjected to the reaction conditions (5 mol% of catalyst **22**, (trifluoromethyl)benzene (TFT) as solvent), gave the unstable 2‐fluoropyridine intermediate **20**, which decomposed in the reaction mixture to the iminium fluoride **21** [40].

**SCHEME 8:**
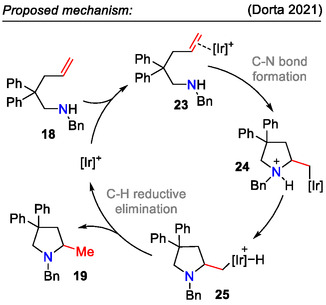
Proposed mechanism for hydroamination of unactivated aminoalkenes **18**.

As mechanistic studies suggested, the above mentioned reaction proceeded by coordination of the olefin functionality in substrate **18** to the catalytically active 14‐electron iridium species via the following steps: 1) C─N bond formation during the amine attack; 2) hydrogen transfer from the ammonium species to the iridium, forming Ir(III)‐hydride intermediates; and lastly, 3) reductive elimination, affording 2‐methyl pyrrolidines **19** (Scheme 8) [40].

**SCHEME 9:**
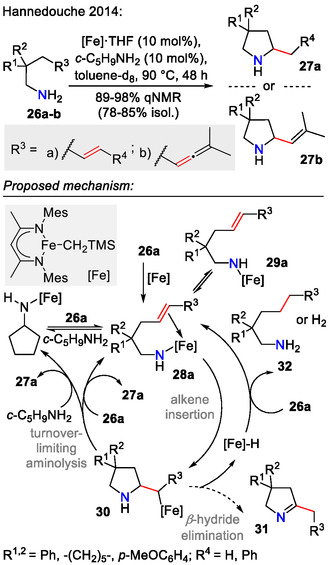
Hydroamination of unprotected primary alkenylamines **26a‐b** employing Fe catalysis.

Hydroamination of unprotected primary alkenylamines **26a** is more challenging, especially if we wish to avoid using noble metal catalysis due to their limited availability, price, and toxicity. One of the few available strategies for sustainable hydroamination of unprotected alkenylamines was reported by Hannedouche et al., who used iron(II) complexes as low‐cost, nontoxic, and abundant metal catalysts for this transformation (Scheme 9). Interestingly, allenes **26b** also participated in this reaction, forming 2‐vinyl substituted pyrrolidines **27b** [41].

Although pyrrolidines **27a‐b** were obtained as major products, the formation of side products **31** and **32** in smaller quantities was also observed. Experimental observations suggested that the reaction selectivity was influenced by the concentration of primary amine present in the mixture. By adding a noncyclizable primary amine—cyclopentylamine (*c*PentNH_2_)—to the reaction mixture, the reaction selectivity was greatly improved, reducing the side product formation to trace amounts. Mechanistic studies, however, stated that the alkene migratory insertion into the Fe─NHR bond happens without the assistance of the additional amine (*c*PentNH_2_). Aminolysis was determined as the turnover‐limiting step of the proposed catalytic cycle (Scheme 9) [41].

**SCHEME 10:**
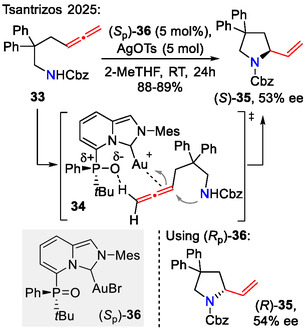
Synthesis of enantioenriched 2‐vinyl pyrrolidine **35** from allene **33** using chiral Au‐catalysts.

Enantioenriched 2‐vinyl pyrrolidines **35** were obtained by the Tsantrizos group, using *P*‐chiral phosphine oxide *N*‐heterocyclic carbene‐Au(I) catalysts (*S*_p_)‐ and (*R*_p_)‐**36** for stereoselective intramolecular hydroamination of allenes **33**. (*S*_p_)‐PO‐NHC‐Au(I) catalyst (*S*_p_)‐**36** afforded the (*S*)‐pyrrolidine **35** in excess (53% ee), whereas (*R*_p_)‐PO‐NHC‐Au(I) catalyst (*R*_p_)‐**36** favored formation of the opposite enantiomer (*R*)‐**35** (55% ee). The observed reaction rates were higher compared with the corresponding NHC‐Au(I) catalysts. The reaction rate acceleration was attributed to the formation of a hydrogen bond between the terminal allene proton and catalyst *P*=O, which provided additional stability to the Au(I)‐allene π‐bond complex in transition state **34** (Scheme 10) [42].

**SCHEME 11:**
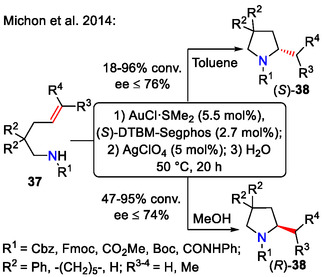
Au‐catalyzed asymmetric alkene **37** hydroamination.

Higher ee (up to 76%) for intramolecular hydroamination of alkenes **37** was achieved using a selected diphosphine binuclear gold(I) chloride complex in a combination with silver salt under mild conditions (Scheme 11). While the control experiments showed that AgClO_4_ by itself cannot facilitate this reaction, it played a key role in generating reactive gold cationic species by abstraction of halides from the neutral gold complex, thus greatly improving reactivity. In several cases, conversions up to 96% could be achieved. The reactivity was influenced by alkene **37** *R* ^3,4^ substituents in the following manner: H (most reactive) >> Me (least reactive). The substituents in C2 position (*R* ^2^) also had a critical effect on conversion: Ph (highest conversion) > ‐(CH_2_)_5_‐ >> H (lowest conversion). Thorpe–Ingold effects, created by bulky *R* ^2^ substituents, also contributed to the stereochemical outcome: ‐(CH_2_)_5_‐ (highest ee) > Ph >> H (lowest ee) [34].

**SCHEME 12:**
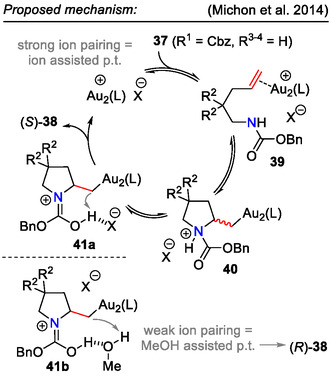
Solvent‐dependent proton transfer mechanism in Au‐catalyzed alkene **37** hydroamination.

Interestingly, by controlling the molecular ion pairs through the solvent polarity, both enantiomers of the product **38** could be obtained. Stronger ion pairing, as observed in toluene, favored the formation of (2*S*)‐stereoisomers (Scheme 12). Methanol as a solvent likely assisted in proton transfer, weakening the ion pairing; thus, the opposite stereoselectivity was obtained [34].

**SCHEME 13:**
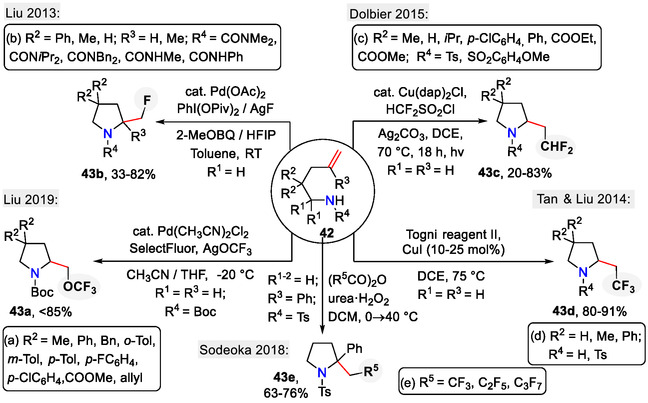
Synthesis of fluorinated pyrrolidines **43**.

**SCHEME 14:**
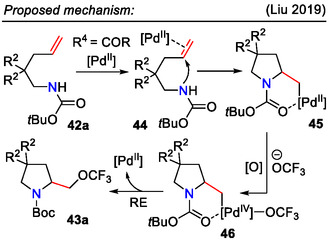
Proposed mechanism for the synthesis of CF_3_O‐group‐containing pyrrolidines **43a**.

The introduction of fluorine‐containing functionalities in the pyrrolidine side chain has gained much interest due to the abundance of fluorinated drugs [43, 44, 45]. Several new cyclization methods, following the *5‐exo‐trig*‐pathway, have been developed for the synthesis of fluorinated pyrrolidines **43a‐e** as summarized in Scheme 13 [46, 47, 48, 49, 50]. Liu and coworkers used palladium‐catalyzed intramolecular oxidative amination of alkenes for the synthesis of CF_3_O‐group‐containing pyrrolidines **43a** (Schemes 13 and 14). In this case, AgOCF_3_ was used as the trifluoromethoxide source and SelectFluor as an oxidant. Regarding the substrate design, the amine protecting group had a significant influence on selectivity between the competing *5‐exo‐trig*‐ and *6‐endo‐trig* cyclization pathways. Following amine protecting groups promoted the *5‐exo‐trig* pathway, leading to pyrrolidines **43a**: Bz (lowest yield) < *N*‐Me_2_NCO < Boc (highest yield). Sulfonamide protecting groups (SO_2_NMe_2_) promoted the competing *6‐endo‐trig‐*pathway instead, affording 3‐CF_3_O‐piperidines as major products [46].

Mechanistic studies confirmed that the above‐mentioned reaction proceeded via the palladacycle intermediate **45** (Scheme 14). The insertion of the CF_3_O group in the pyrrolidine side chain was likely the result of reductive elimination from the Pd(IV)‐intermediate **46** [46].

In a mechanistically analogous process, the Liu group achieved aminofluorination of similar substrates **42b** by using Pd(OAc)_2_ as a catalyst and the AgF/ PhI(OPiv)_2_ system as a fluorinating agent, affording in the side chain monofluorinated pyrrolidines **43b** (Scheme 13) [47].

**SCHEME 15:**
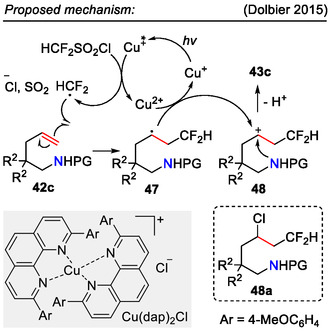
Proposed mechanism for the synthesis of CHF_2_ functionality bearing pyrrolidines **43c**.

For the incorporation of the CHF_2_ functionality into the pyrrolidine side chain, the Dolbier group used the Cu(dap)_2_Cl photoredox catalyst instead (Scheme 13). In the proposed mechanism (Scheme 15), the photoexcited Cu(I)‐catalyst generated the HCF_2_ radical by single electron reduction of HCF_2_SO_2_Cl. The HCF_2_ radical reacted with the alkene **42c**, forming radical intermediate **47**. Following oxidation afforded the carbenium ion intermediate **48**, which was then quenched by the internal amide nucleophile, forming the pyrrolidine ring **43c** with a CHF_2_ functionality in the side chain. Addition of Ag_2_CO_3_ to the reaction mixture ensured chemoselectivity by suppressing the formation of chlorinated side products **48a** [48].

**SCHEME 16:**
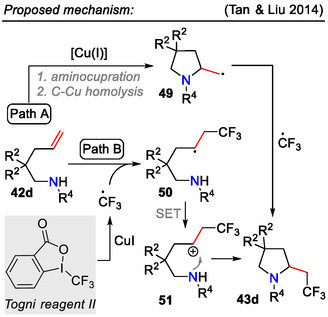
Proposed mechanism for the synthesis of CF_3_‐group‐containing pyrrolidines **43d**.

In another copper‐catalyzed process, Tan and Liu obtained CF_3_‐containing pyrrolidines **43d**, wherein Togni II reagent served as CF_3_ radical source to furnish an intramolecular aminotrifluoromethylation sequence in unactivated alkenes **42d** (Scheme 13). Two mechanistic pathways were proposed for the aminotrifluoromethylation reaction (Scheme 16). In pathway A, Cu(I)‐catalyzed aminocupration of the alkene **42d**, followed by homolytic cleavage of the C─Cu bond, afforded the primary radical **49**, which recombined with the CF_3_ radical, forming the fluorinated pyrrolidine derivative **43d**. In pathway B, the CF_3_ radical reacted with the alkene **42d** to form the radical intermediate **50**, which was further oxidized to the carbenium ion **51** via a single electron transfer (SET) process. Following C─N bond formation via amide nucleophilic attack provided the cyclic product **43d** [49].

**SCHEME 17:**
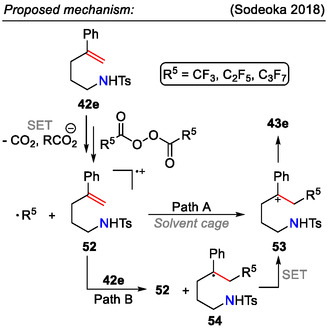
Proposed mechanism for transition‐metal‐free synthesis of perfluoroalkylated pyrrolidines **43e**.

As an alternative to the transition metal‐catalyzed approaches, Sodeoka demonstrated a unique reactivity pattern between styrenes **42e** and bis(perfluoroacyl) peroxides, generated in situ from perfluoro acid anhydrides (Schemes 13 and 17). Through a radical‐carbenium ion cascade reaction, perfluoroalkylated pyrrolidines **43e** were obtained [50].

**SCHEME 18:**
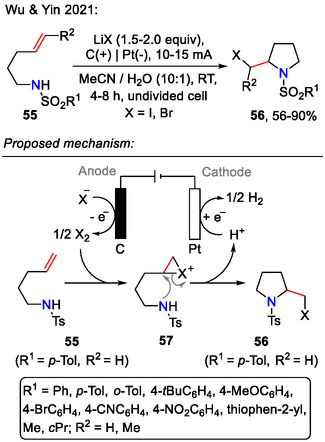
Electrochemical synthesis of halogenated pyrrolidines **56**.

Other halides (I, Br) were introduced into the pyrrolidine side chain via an electrochemical halo‐amination approach by Wu and Yin, employing anodic oxidation to generate molecular I_2_ and Br_2_ species in situ from the corresponding lithium salts (Scheme 18). The alkene **55** was activated by formation of the iodonium/bromonium intermediate **57**, followed by intramolecular nucleophilic substitution via amide attack to afford the pyrrolidine **56**, bearing a halogen atom in the side chain. In contrast to the previously discussed intramolecular amination methods, the electrochemical approach was mild and did not rely on external oxidants or transition metal catalysis. Furthermore, the synthetic utility of electrochemical halo‐amination was demonstrated by the late‐stage functionalization of drug molecules *celecoxib* and *valdecoxib* [51].

**SCHEME 19:**
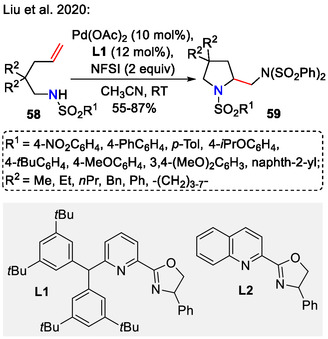
Pd‐catalyzed diamination of unactivated olefins.

Introduction of an additional amine functionality into the pyrrolidine side chain was enabled by Liu and coworkers, who developed a ligand‐controlled system for Pd‐catalyzed diamination of unactivated olefins **58** (Scheme 19). The choice of palladium ligand was crucial in determining the cyclization regioselectivity—the bulky pyox ligand (**L1**) favored the *5‐exo‐*cyclization pathway to give pyrrolidines **59**. The less hindered quinox ligand (**L2**), however, promoted the *6‐endo‐*cyclization pathway, leading to piperidine rings. The reaction presumably proceeded through a reversible aminopalladation step, following oxidative addition of R_2_N‐F (rate‐limiting step) and C─N bond formation via a reductive elimination pathway [52].

**SCHEME 20:**
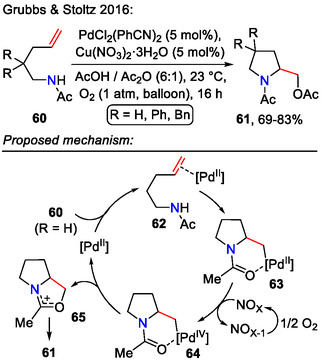
Pd‐catalyzed aminoacetoxylation of olefins **60**.

Under aerobic conditions, palladium‐catalyzed alkene **60** difunctionalization led to side chain acetoxylation, as demonstrated by Grubbs and Stoltz (Scheme 20). Oxidation of the alkylpalladium(II) intermediate **63** with O_2_ as the only oxidant was kinetically challenging; therefore, facile *β*‐hydride elimination was an issue. To access the high‐valent palladium(IV) intermediate **64** more easily, catalytic NO_*X*_ species were added to serve as electron transfer mediators. While Cu(NO_3_)_2_·3H_2_O afforded the highest pyrrolidine **61** yields (up to 83%), nitrites, such as AgNO_2_ or *i*BuONO, were also applicable. After Pd oxidation (II→IV), C─O bond forming reductive elimination and acetolysis of the formed cationic intermediate **65**, afforded the aminoacetoxylation product **61** [53].

**SCHEME 21:**
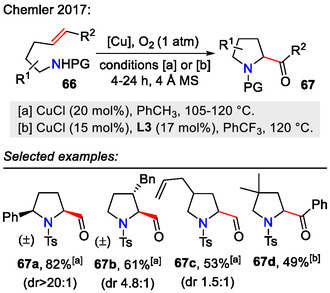
Copper‐catalyzed aminooxygenation for the synthesis of 2‐formyl and 2‐ketopyrrolidines.

Copper(I) catalysis under aerobic conditions, on the other hand, led to the synthesis of 2‐formyl and 2‐ketopyrrolidines **67**, as demonstrated in the aminooxygenation approach by the Chemler group (Scheme 21). Depending on the substitution pattern, moderate to high diastereoselectivity for the formed pyrrolidines **67** was observed, for example, 2,5‐disubstituted pyrrolidines were obtained with excellent *cis*‐selectivity (dr > 20:1). Interestingly, 2‐formylpyrrolidines (*R* ^2^= = H) could be oxidized further to pyrrolid‐2‐ones **77** by slightly adjusting the reaction conditions, that is, adding the bis(oxazoline) ligand **L3** and an external base (DABCO). For 2‐ketopyrrolidines (*R* ^2^ = Ph), the reaction stopped at product **67**, even in the presence of ligand **L3** [54].

**SCHEME 22:**
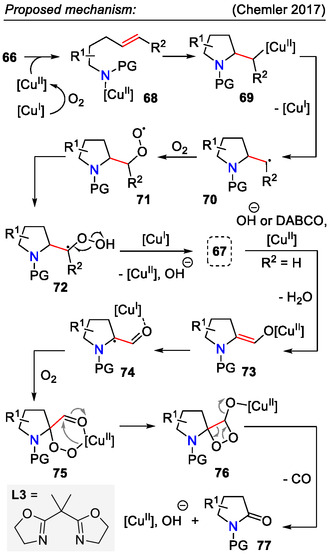
Proposed mechanism for copper‐catalyzed aminooxygenation of alkene **66**.

In the hypothesized aminooxygenation mechanism (Scheme 22), the copper(I) catalyst was oxidized to copper(II) in an O_2_ atmosphere. Next, alkene **66** *cis*‐aminocupration occurred, forming the unstable intermediate **69**, which underwent C‐Cu homolysis, liberating the secondary radical species **70**. Molecular O_2_ reacted with the formed radical to form the 2‐formyl / ketopyrrolidine structure **67**. Unless isolated, formylpyrrolidines **67** (*R* ^2^ = H) could react further with an in situ‐formed hydroxide or external base (DABCO) to form an enolate intermediate **73**. Further reaction with molecular oxygen afforded peroxy intermediates **75** and then **76**. Subsequent oxidative C─C bond cleavage resulted in pyrrolid‐2‐one **77** formation [54].

**SCHEME 23:**
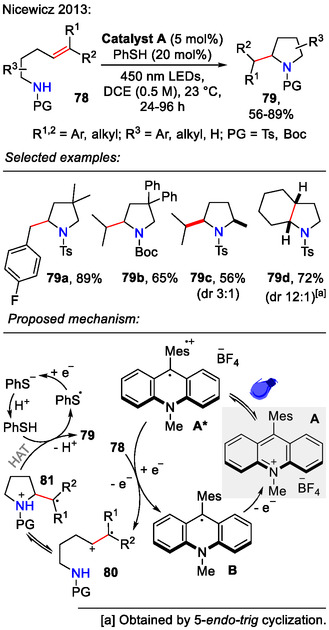
Organic photoredox system‐catalyzed hydroamination of alkenes **78**.

A metal‐free approach was demonstrated by the Nicewicz group, who performed direct anti‐Markovnikov hydroamination of unsaturated amines **78** (Scheme 23). It required visible‐light irradiation and catalytic quantities of 9‐mesityl‐10‐methylacridinium tetrafluoroborate (photocatalyst **A**; 5 mol%). Thiophenol (20 mol) was added as a hydrogen atom donor. In substrates where the 5‐*exo‐trig* cyclization pathway was not feasible due to carbon chain length, 5‐*endo‐trig* cyclization occurred, as demonstrated by example **79d**, where the bicyclic pyrrolidine structure was obtained with excellent diastereoselectivity. (dr 12:1). For 2,5‐disubstituted pyrrolidines, the *cis*‐selectivity was less pronounced (e.g., **79c**, dr 3:1). Electron‐rich substrates (PG = Bn, H) afforded poor yields (5%–15%) due to being prone to oxidation. Ts‐ or Boc‐protected amines **78**, however, afforded pyrrolidines **79** with 56%–89% yields [55].

For the mechanistic rationale (Scheme 23), a catalytic cycle was proposed, where the photoexcited catalyst A* oxidized the olefin **78** to a radical cation **80**. Subsequent anti‐Markovnikov addition of the amine gave intermediate **81**. The catalytic cycle was finalized by H‐atom transfer from PhSH to obtain pyrrolidine **79** after proton loss. The liberated thiyl radical (PhS⋅) was proposed to assist in reoxidation of the acridine radical **B**, thus facilitating the regeneration the photocatalyst **A** [55].

**SCHEME 24:**
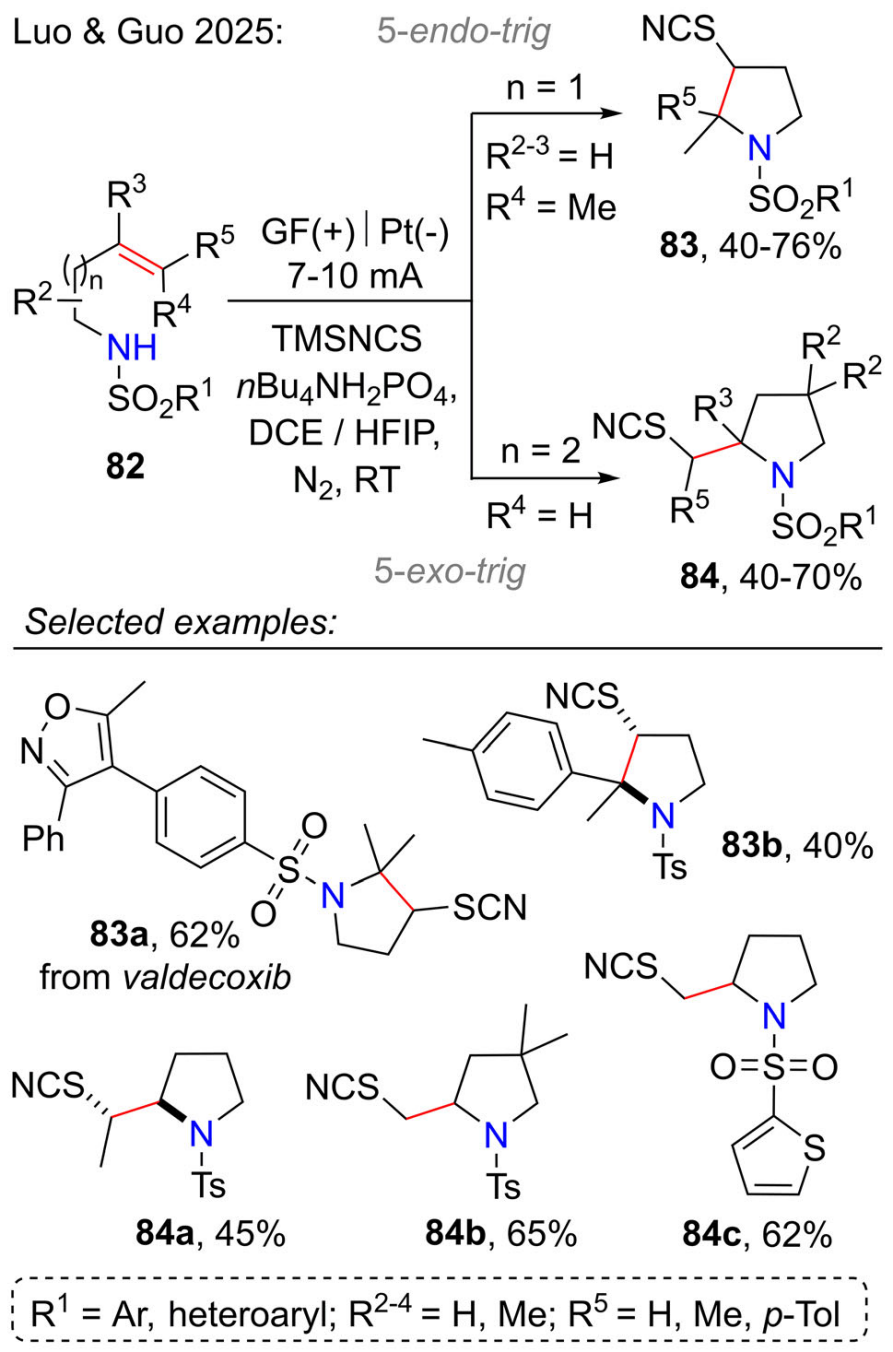
Electrochemical aminothiocyanation of olefins **82** for the synthesis of functionalized products **83** and **84**.

To conclude with 5‐*exo*‐*trig* cyclizations, an electrochemical aminothiocyanation method was developed for sulfonamide‐containing unactivated olefins **82**. Depending on the carbon chain length, cyclization occurred either via the 5*‐endo‐trig* or 5‐*exo‐trig* pathway to afford the thiocyanate functionality‐bearing pyrrolidines **83** or **84**, respectively (Scheme 24). Electrochemical aminothiocyanation offered the advantage of mild reaction conditions, as well as good regioselectivity. Some examples for late‐stage functionalization of bioactive molecules were also demonstrated, such as *valdecoxib* and *celecoxib*. This reaction was compatible with various functional groups in the aryl and heteroaryl (*R* ^1^) sulfonamide ring: OMe, OCF_3_, CF_3_, Me, *t*Bu, F, Cl, and Br. In substrates, where the formation of two stereogenic centers was possible, aminothiocyanation proceeded diastereoselectively to afford the *anti*‐products**—83b** and **84a** [56].

**SCHEME 25:**
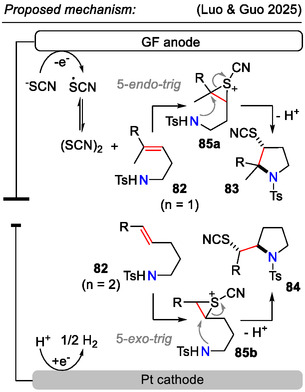
Mechanistic proposal for electrochemical aminothiocyanation of olefins **82**.

Mechanistic investigations for the electrochemical aminothiocyanation approach suggested that the cyclization step might proceed via a sulfonium ion **85a‐b** (Scheme 25). Ring opening by nucleophilic attack via the 5*‐endo‐trig* or 5‐*exo‐trig* pathway resulted in thiocyanation of the pyrrolidine C3‐position or the side chain, respectively [56].

### 2.2 5‐*Endo‐Trig*‐Cyclization

**SCHEME 26:**
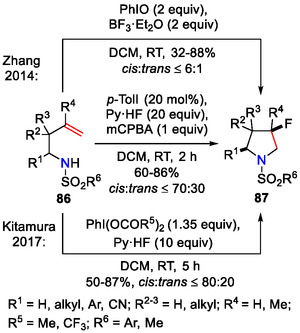
3‐Fluoropyrrolidine **87** synthesis.

Zhang and coworkers combined PhIO‐facilitated cyclization of homoallylic amines **86** with BF_3_·Et_2_O as a fluoride source to furnish a new pathway towards 3‐fluoropyrrolidines **87** (Scheme 26). In the case of 2‐alkyl or aryl substituted homoallylic amines (*R* ^1^ = Et, *t*Bu, Ph, 4‐*t*BuC_6_H_4_, 3‐CF_3_C_6_H_4_; *R* ^2−4^ = H), the fluorinated products **87** were obtained with moderate diastereoselectivity, varying from dr 4.3:1 to 6.0:1 (*cis*/*trans*) [57].

Similarly, 3‐fluoropyrrolidines **87** were synthesized by Kitamura and coworkers using a reagent system of PhI(OAc)_2_ and Py·HF (Scheme 26) [58]. Other hypervalent iodine reagents, such as PhI(OCOCF_3_)_2_, were also applicable in this reaction. In the catalytic version, also explored by Kitamura, 20 mol% of *p*‐iodotoluene was combined with Py·HF as a fluoride source and *m*CPBA as an oxidant to afford 3‐fluoropyrrolidines **87** with yields up to 86% [58].

**SCHEME 27:**
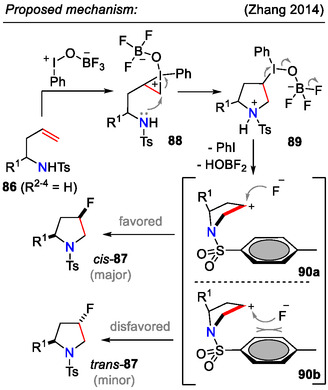
Mechanistic proposal for the PhIO/BF_3_·Et_2_O facilitated aminofluorination of olefins **86**.

Based on experimental evidence, a mechanism involving a carbenium ion intermediate was proposed for the iodosobenzene‐mediated transformation **86→87** (Scheme 27). First, iodine(III) intermediate was formed by activation of PhIO with BF_3_·Et_2_O. The iodine(III) reagent then reacted with the alkene **86** (*R* ^2–4^ = H) to give an iodonium intermediate **88**. Subsequent nucleophilic attack of the amino group closed the pyrrolidine ring **89**. The hypervalent iodine(III) intermediate then underwent reductive elimination to afford the cyclic carbocation **90a‐b**, which was quenched by the fluoride anion to afford the fluorinated product **87** (*R* ^2–4^ = H). The preference for *cis*‐selectivity could be explained by the steric hindrance from the sulfonyl group, where one side of the pyrrolidine ring is blocked against the nucleophilic attack of the fluoride ion [57].

**SCHEME 28:**
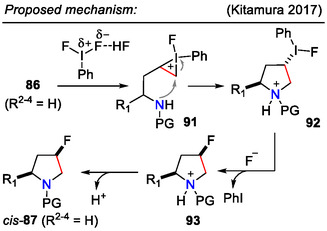
Mechanistic rationale for aminofluorination of olefins **86**, involving an S_*N*_2‐type step for C─F bond formation.

A slightly different interpretation of the mechanism was later offered by Kitamura and coworkers (Scheme 28), where the C─F bond was formed by S_*N*_2‐type substitution during the fluoride ion attack on intermediate **92**. The preferential formation of the *cis‐* **87** isomer was explained by the formation of the sterically favored intermediate *trans*‐**92**. Mechanistic studies suggested that the reaction proceeded via the iodonium intermediate **91** [58].

**SCHEME 29:**
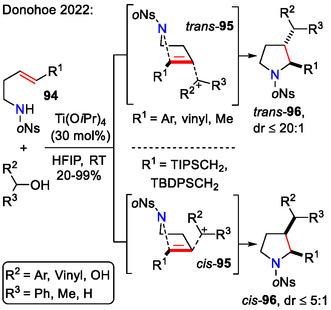
Titanium‐catalyzed diastereoselective synthesis of 2,3‐disubstituted pyrrolidines **96**.

Other than hypervalent iodine(III)‐mediated cyclizations, some new Lewis acid‐catalyzed methods were explored as well. One of them was a highly diastereoselective Ti(O*i*Pr)_4_‐catalyzed approach towards 2,3‐substituted pyrrolidines **96**, which was offered by the Donohoe group (Scheme 29). The reaction was triggered by the generation of a cationic intermediate from allylic or benzylic alcohols, followed by simultaneous C─C and C─N bond formation with the alkenylamide **94**. The size of substituent *R* ^1^ was crucial in determining 2,3‐*cis‐* or *trans‐*product formation. Bulky TIPSCH_2_‐ or TBDPSCH_2_‐groups promoted *cis*‐product formation, possibly due to creating a steric clash with the incoming allyl/benzyl cation in the transition state **95**. Interestingly, when allylic alcohols bearing two different terminal substituents were used, partial regiocontrol could be achieved. It was presumably due to a preferential attack at the least hindered end of an unsymmetrical allyl cation intermediate [59].

**SCHEME 30:**
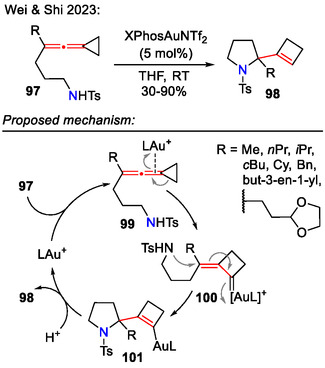
Gold‐catalyzed cascade cyclization of vinylidenecyclopropanes **97**.

Wei and Shi reported a gold(I)‐catalyzed cascade cyclization of *N*‐nucleophile tethered vinylidenecyclopropanes **97**, resulting in pyrrolidine scaffolds **98**, bearing a cyclobutene moiety in the side chain (Scheme 30). This transformation occurred via ring expansion of the cyclopropane unit, followed by gold carbene‐induced vinylogous nucleophilic addition in 5‐*endo‐trig* fashion. Regarding the applicable substrates for the gold‐catalyzed cyclization, apart from alkyl and benzyl substituted (*R* = alkyl, Bn) allenes **97**, some more complex substrates **97**, functionalized with a terminal olefin or 1,3‐dioxolan‐2‐yl group in the side chain (R = but‐3‐en‐1‐yl or 2‐(1,3‐dioxolan‐2‐yl)ethyl) also afforded the desired product **98**, whereas substrates bearing aromatic groups (*R* = 4‐ClC_6_H_4_) did not. Lower yields were obtained for sterically hindered alkyl substituents (*R* = *i*Pr, Cy) [60].

### 2.3 5‐*Exo‐Dig*‐Cyclization

**SCHEME 31:**
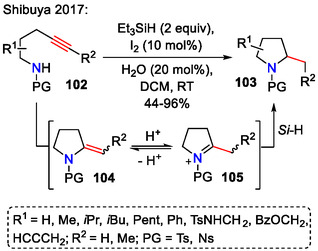
Pyrrolidine **103** synthesis from alkynes **102** by a hydroamination/reduction sequence.

While there are several transition‐metal‐free methods for intramolecular amination of unactivated alkenes, the same for unactivated alkynes has rarely been reported. The inherent instability of a vinyl cation intermediate was one of the obstacles, preventing further development of transition‐metal‐free procedures. Shibuya and coworkers addressed this problem by using a silane‐iodine system for the intramolecular hydroamination/reduction of unactivated alkynes **102** (Scheme 31) [61].

The above‐mentioned reaction likely proceeded through an exo‐cyclic enamine intermediate **104**, which rearranged to provide an iminium intermediate **105**. Further reduction by Et_3_SiH produced pyrrolidines **103**. High diastereoselectivity was achieved under these conditions—dr 10:1 (*cis*/*trans*) for 2,4‐disubstituted pyrrolidines **103** and dr 23:1 (*cis*/*trans*) for 2,5‐disubstituted products **103** [61].

### 2.4 5‐Endo‐Dig‐Cyclization

**SCHEME 32:**
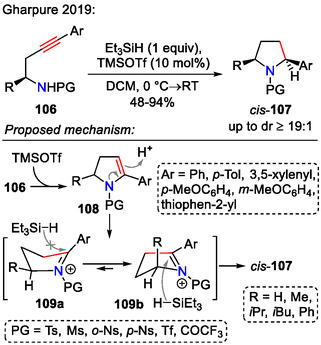
Diastereoselective synthesis of *cis*−2,5‐disubstituted pyrrolidines **107** from chiral alkynes **106**.

Pyrrolidine synthesis via the 5‐*endo‐dig*‐cyclization pathway has been studied more extensively in recent years than its *exo*‐counterpart. The Gharpure group reported a TMSOTf‐mediated reductive hydroamination of chiral homopropargylic amines **106** for the diastereoselective synthesis of 2,5‐disubstituted pyrrolidines **107** (Scheme 32). Reduction of the intermediate iminium ion with triethylsilane from the least hindered face (transition state **109b**) favored the formation of *cis*−2,5‐disubstituted pyrrolidines **107** (up to dr ≥ 19:1) [62].

**SCHEME 33:**
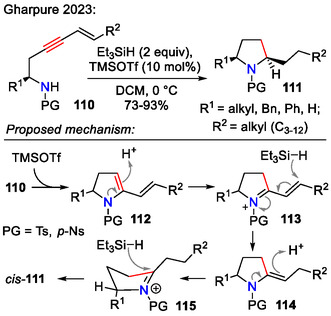
Synthesis of 2,5‐disubstituted pyrrolidines **111**, bearing long hydrophobic side chains.

The same Et_3_SiH / TMSOTf system, when used for reductive hydroamination of alkenynes **110**, bearing a pendant sulfonamide, afforded pyrrolidines **111**, bearing longer hydrophobic chains in the C2‐site (Scheme 33). The alkenyne substrate **110** was conveniently prepared in one step via *Sonogashira* coupling between the corresponding monosubstituted terminal alkyne and vinyl halide. This cyclization approach was employed in the synthesis of various pyrrolidine alkaloids, namely (±)‐*cis*‐225H, (±)‐*epi*‐197B, (±)‐*epi*‐225C, and (±)‐bgugaine [63].

**SCHEME 34:**
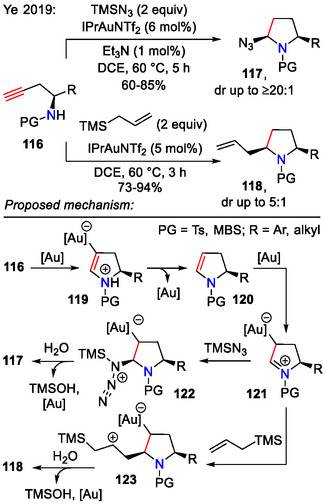
Gold‐catalyzed hydroamination of chiral homopropargyl sulfonamides **116**.

Gold catalysis also enabled intramolecular hydroamination of chiral homopropargyl sulfonamides **116**, as demonstrated by Ye and coworkers (Scheme 34). The reaction proceeded via the 2,3‐dihydropyrrole intermediate **120**, which could undergo further azidation or allylation to yield the corresponding enantioenriched 2,5‐disubstituted pyrrolidines **117** and **118** [64].

**SCHEME 35:**
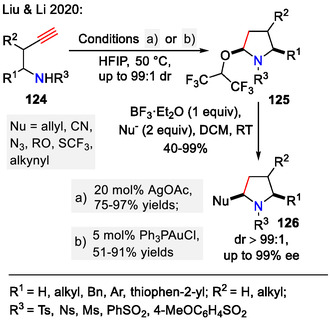
Cyclization of homopropargylic sulfonamides **124** with further side chain derivatization.

Similarly, homopropargylic sulfonamides **124** could be activated using a silver catalyst (20 mol% AgOAc), inducing the *5‐endo‐dig*‐type cyclization (Scheme 35). Gold catalysis also facilitated this transformation and with lower catalyst loading (5 mol% Ph_3_PAuCl). Using the weakly nucleophilic HFIP as solvent, isolatable *N*, *O*‐acetals **125** as intermediates were obtained. Notably, nearly all the 2‐hexafluoroisopropoxypyrrolidine products **125** were obtained in the *cis*‐configuration. The only exception were *N*‐4‐nitrobenzenesulfonyl‐substituted products (*R* ^1^ = Ph, *R* ^2^ = H, *R* ^3^ = Ns), where the *trans*‐isomer **125** was obtained as the main product (dr *= *1:6.6 *cis*/*trans*), possibly due to the electronic effects on the sulfonyl aromatic ring. Additionally, complete chirality retention was observed in reactions with enantiopure homopropargylic sulfonamides **124**, which afforded *cis*−2,5‐disubstituted pyrrolidines **125** with high diastereoselectivity (up to 99:1 dr). Further reactions of the obtained *N*, *O‐*acetals were investigated by the addition of external nucleophiles. The (CF_3_)_2_CHO‐ substituent acted as a good leaving group, and 2,5‐bifunctionalized pyrrolidines **126** were obtained. In this manner, several new C─C and C‐Het (Het = *N*, *O*, *S*) bonds were constructed. Moreover, applications of this approach were tested for the synthesis of the pharmaceutical intermediate of *trandolapril* **126** (*R* ^1,2^ = ‐(CH_2_)_4_‐, *R* ^3^ = Ts, Nu = CN) from the corresponding homoallylic sulfonamide **124** (*R* ^1,2^ = ‐(CH_2_)_4_‐, *R* ^3^ = Ts) in 75% yield over 2 steps. Also, successful late‐stage functionalization of *β‐estradiol* was achieved using this approach [65].

**SCHEME 36:**
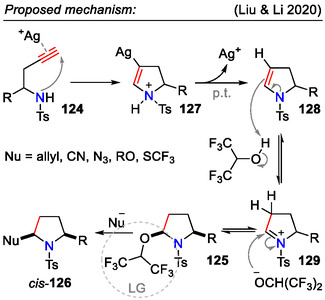
Mechanistic proposal for silver‐catalyzed cyclization of homopropargylic sulfonamides **124**.

Mechanistic studies suggested a reaction pathway involving dihydropyrrole intermediate **128** (Scheme 36). Control experiments demonstrated that the hydroalkoxylation step with HFIP proceeded with no requisition of the Ag(I) catalyst, but at a slower rate, for example, 3.5 h in the presence of AgOAc and 48 h without the catalyst [65].

## 3 Tandem Annulation Strategies Incorporating Intramolecular C─N Bond Formation as a Key Step

**SCHEME 37:**
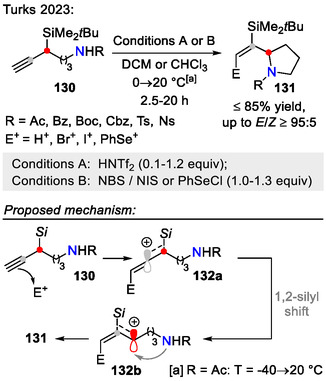
Pyrrolidine **131** synthesis from propargyl silanes **130** via the 1,2‐silyl shift.

Instead of direct intramolecular amination, propargylic silicon‐containing alkynes can undergo cationic rearrangement to form stabilized allylic cations before participating in the key C─N bond formation step. This was demonstrated in a recent example by the Turks group, where propargyl silanes **130**, containing a tethered amide nucleophile, were heterocyclized to afford functionalized olefin side chain containing pyrrolidines **131** (Scheme 37). Upon electrophilic activation of the propargyl silane moiety, the formed vinyl cation **132a** rearranged to the more stabilized allyl cation **132b** via the 1,2‐silyl shift. The allylic cation **132b** was then quenched by the internal amine, forming the pyrrolidine ring **131** [66]. This transformation was possible due to the *β*‐carbocation‐stabilizing properties of silicon [67]. In addition to pyrrolidine ring formation, a highly stereodefined olefin side chain was introduced in the pyrrolidine C(2)‐position. Further utility of the vinyl halide‐containing reaction products was demonstrated by participation in Suzuki‐Miyaura coupling reactions. As for the possibilities to displace the silyl group, electrophilic substitution using NIS in 2,2,2‐trifluoroethanol was successfully performed, while preserving the double‐bond geometry [66].

**SCHEME 38:**
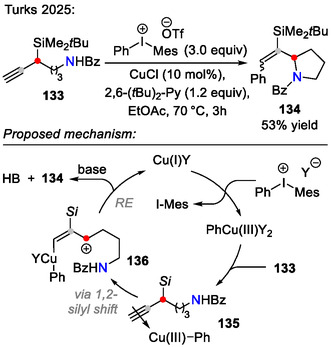
Copper‐catalyzed arylation‐cyclization sequence for propargyl silanes **133**.

Similarly, a copper‐catalyzed arylation‐ring closure strategy was developed for direct synthesis of pyrrolidines **134**, bearing a functionalized styrene side chain, utilizing diaryl‐*λ* ^3^‐iodanes and propargyl silanes **133** as starting materials (Scheme 38). Although this method was primarily designed for terminal alcohol group‐containing silanes (C─O bond formation), benzamide **133** also underwent a similar arylation‐cyclization sequence, affording pyrrolidine **134** [68].

For the above‐mentioned transformation, a Cu(I/III) catalytic cycle was proposed (Scheme 38), where Cu(I) reacted with the diaryl‐*λ* ^3^‐iodane to generate the strongly electrophilic Ar‐Cu(III) species, which activated the propargylic system to induce 1,2‐silyl migration. The afforded allylic cation intermediate **136** was trapped by the internal amide group to form the pyrrolidine ring **134**. Lastly, reductive elimination regenerated the Cu(I) catalyst [68].

**SCHEME 39:**
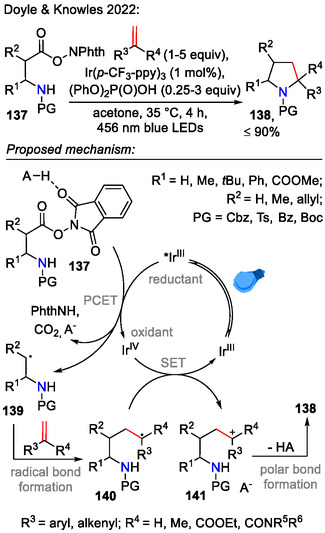
Iridium‐catalyzed annulation strategy, involving a polar C─N bond formation step.

Multistep synthesis of starting materials, bearing both the amide and alkene/alkyne functionalities, is the general drawback in the so far discussed intramolecular C─N bond strategies. Tandem processes, such as recent radical redox annulation strategies, aim to streamline the synthesis of pyrrolidine **138** by offering a direct route from alkenes and *N*‐hydroxyphthalimide esters **137**. In one example by Doyle and Knowles, an iridium(III)‐catalyzed photocatalytic approach was employed for annulation of alkenes with bifunctional reagents **137**, containing *N*‐hydroxyphthalimide ester (NHPI) and tethered 2° amide functionalities (Scheme 39). Visible light irradiation (456 nm) activated the iridium(III) photocatalyst. This initiated single‐electron reduction of the NHPI ester **137**, leading to fragmentation into radical species **139**. The latter reacted with an external alkene in anti‐Markovnikov fashion, forming intermediate **140**, containing both the radical and tethered amide functionalities. Single‐electron oxidation provided the strategic intermediate **141**, which participated in the polar C─N bond formation step, affording pyrrolidine **138**. Although the substrate scope was limited to aryl alkenes and dienes (cyclic and acyclic), this reaction found some interesting applications in the late‐stage derivatization of pharmaceutical compounds, such as *ezetimibe*, *febuxostat*, and *amoxepine* [69].

**SCHEME 40:**
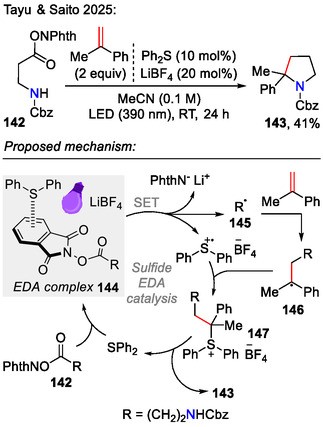
Visible‐light‐driven synthesis of pyrrolidines **143**, utilizing a phthalimide ester/sulfide EDA system.

Similarly, under visible‐light‐driven conditions, an electron donor–acceptor (EDA) system, consisting of the *N*‐hydroxyphthalimide ester **142** and diphenyl sulfide (electron donor), was used to induce phthalimide ester fragmentation via a SET process to afford reactive radical species **145** (Scheme 40). Radical **145** was captured by an olefin, followed by the addition of diphenyl sulfide cation radical to form the sulfonium species **147**. Intramolecular cyclization by *N*‐nucleophile attack afforded the pyrrolidine **143** scaffold. Lewis acidic lithium ions were added to enhance the interactions within the EDA complex. This visible‐light‐driven method was compatible with water and air, signifying the overall robustness of this method [70].

**SCHEME 41:**
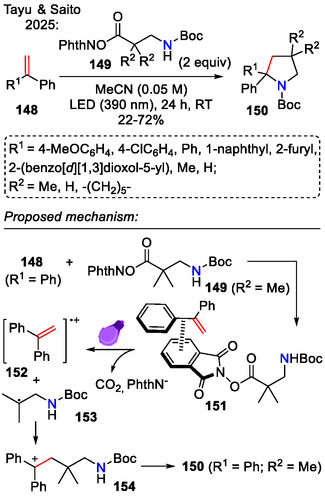
Visible‐light‐induced aminoalkylation of alkenes **148**.

In a mechanistically similar approach, the carbenium ion intermediate **154** was generated under catalyst‐free conditions from styrenes **148** and *N*‐hydroxyphthalimide esters **149**, bearing a tethered carbamate (Scheme 41). It was suggested that in this reaction, styrene formed an EDA complex **151** with the phthalimide ester. LED irradiation caused a SET process, followed by fragmentation to a radical cation **152** and an alkyl radical **153**. Their recombination to cationic intermediate **154**, followed by intramolecular cyclization, afforded the pyrrolidine **150** scaffold. Pyrrolidine **150** yields were generally higher, with aromatic or heteroaromatic *R* ^1^ substituents (up to 72%), whereas aliphatic *R* ^1^ substituents (Me, H) gave poor yields (22%–24%). While the catalyst‐free visible‐light induced method, which proceeded via an electron donor–acceptor complex, was more sustainable, much higher yields were obtained with iridium catalysis. For comparison, the synthesis of *tert*‐butyl 4,4‐dimethyl‐2,2‐diphenylpyrrolidine‐1‐carboxylate **150** (*R* ^1^ = Ph, *R* ^2^ = Me) in accordance with the conditions presented in Scheme 41, afforded the product in 42% yield. Alternatively, performing the reaction using [Ir(dF(CF_3_)ppy)_2_bpy]PF_6_ (1 mol%), *i*Pr_2_S (10 mol%) in MeCN (0.05 M) under LED (390 nm) at room temperature for 24 h afforded the same product **150** (*R* ^1^ = Ph, *R* ^2^ = Me) in quantitative yield [71].

**SCHEME 42:**
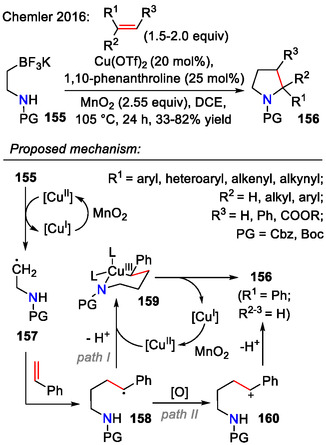
Copper‐catalyzed annulation of *β*‐aminoethyl trifluoroborates **155** with styrenes/dienes for the synthesis of pyrrolidines **156**.

In a non‐photocatalytic approach, featuring Cu(II)‐catalyzed coupling of potassium *β*‐aminoethyl trifluoroborates **155** with styrenes and dienes, 2‐aryl‐ and 2‐vinylpyrrolidines **156** were obtained (Scheme 42). Both 1,2‐disubstituted and 1,1‐disubstituted vinyl arenes participated in this reaction. It was also shown that alkenynes could participate in this reaction, producing 2‐alkynyl‐substituted pyrrolidines **156**. This intermolecular coupling reaction occurred via a copper‐catalyzed/MnO_2_ mediated stepwise oxidative coupling sequence, where Cu(II)‐catalyzed oxidation of the alkylborane **155** liberated the alkyl radical **157**, which added to styrene to produce the stabilized benzylic radical **158**. Next, recombination with Cu(II) afforded the alkylcopper(III) intermediate **159**, which underwent further reductive elimination (path I). Alternatively, the stabilized radical **158** could undergo oxidation by MnO_2_ to the carbenium ion **160**, which is prone to undergo nucleophilic attack by the pendant amide, resulting in pyrrolidine **156** formation (path II) [72].

**SCHEME 43:**
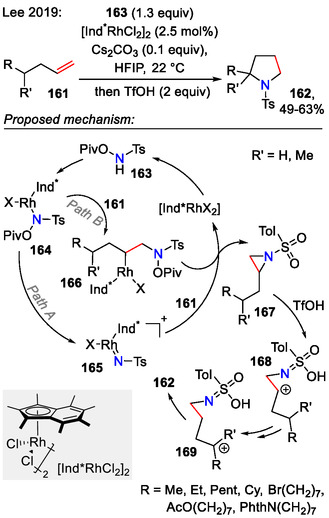
Rhodium‐catalyzed olefin **161** aziridination/cyclization sequence for the synthesis of pyrrolidines **162**.

Later, a Rh(III)‐catalyzed approach was developed, involving a possible rhodium nitrene **165** formation step from 4‐methyl‐*N*‐(pivaloyloxy)benzenesulfonamide (**163**) and subsequent aziridination of olefin **161** (Scheme 43; path A). An alternative pathway for aziridine intermediate **167** formation was also plausible, featuring a migratory insertion step between alkene **161** and intermediate **164**, with a subsequent C─N bond formation step in concerted fashion and N─O bond cleavage (path B). Aziridine **167** ring expansion was succeeded by a combination of sequential hydride shifts and elimination/protonation pathways until the carbenium ion was quenched at the C4 position, forming the pyrrolidine product **162** [73].

## 4 Nucleophilic Addition to the Carbonyl Group

Intramolecular amination of carbonyl compounds is a well‐established C─N bond formation strategy. The majority of recent studies has focused on its practical applications in natural product synthesis or reductive amination strategies, aiming to simplify the substrate design, as scaffolds, bearing both the carbonyl moiety and an internal *N*‐nucleophile, require multistep synthesis to attain.

**SCHEME 44:**
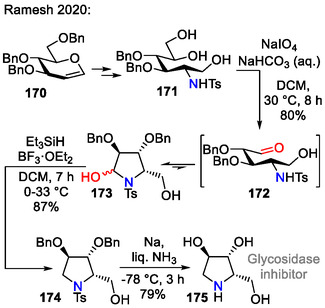
Synthetic route toward 1,4‐dideoxy‐1,4‐imino‐*L*‐xylitol (**175**).

Ramesh and coworkers applied intramolecular amination of aldehyde **172** as the pyrrolidine ring‐formation step in the total synthesis of 1,4‐dideoxy‐1,4‐imino‐*L*‐xylitol (**175**)—a potential glucosidase inhibitor (Scheme 44). Before the C─N bond formation step, the aldehyde **172** was obtained by oxidative cleavage of vicinal diol **171**. Aldehyde **172** heterocyclization afforded the hemiaminal **173**, which was further deoxygenated to pyrrolidine **174** with Et_3_SiH in the presence of BF_3_⋅OEt_2_. Deprotection under Birch conditions afforded 1,4‐dideoxy‐1,4‐imino‐*L*‐xylitol (**175**) [74].

**SCHEME 45:**
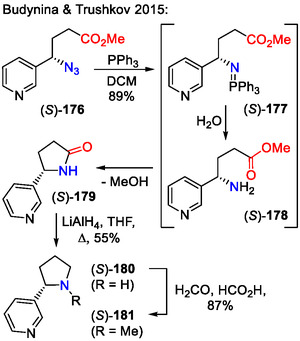
Synthetic route toward (‐)‐nicotine (**181**).

Budynina and Trushkov included a chiral *γ*‐azidoester **176** amination step into the synthetic route toward (‐)‐nicotine (**181**) – a pyrrolidine ring containing alkaloid (Scheme 45). The *γ*‐azidoester **176** was obtained by ring‐opening of cyclopropanes with the azide ion in an S_*N*_2‐like reaction. Reduction of the azido group in starting material **176** under Staudinger conditions, followed by cyclization, afforded the lactam scaffold **179**. The latter was reduced with LiAlH_4_ and methylated to produce (‐)‐nicotine (**181**) [75].

**SCHEME 46:**
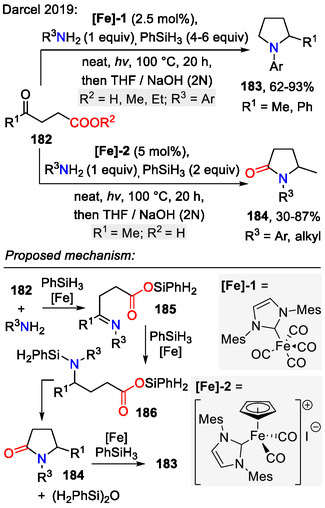
Reductive amination of levulinic acid **182** and its esters.

Darcel and coworkers employed silane RSiH_3_ as a mild reducing agent for the reductive amination of levulinic acid **182** (*R* ^1^ = Me, *R* ^2^ = H) and its esters (Scheme 46). The reaction was catalyzed by an NHC–iron complex: Fe(CO)_4_(*IMes*) (**[Fe]‐1**). In the proposed reaction mechanism, oxocarboxylic acid **182** (*R* ^2^ = H) condensed with aniline R^3^NH_2_, forming the imine intermediate **185**, and silyl derivatization of the carboxylic acid incorporated the O‐Si moiety. Next, iron‐catalyzed hydrosilylation led to the silyl amine **186** and subsequent ring closure by transamidation afforded the pyrrolid‐2‐one intermediate **184**. The latter was reduced to the pyrrolidine **183** in another hydrosilylation event. A few additional details should be pointed out: 1) visible light was crucial for generation of the active 16 electron [Fe^0^]‐species, required to promote the silane oxidative addition step; 2) depending on the employed iron catalyst, the reaction proceeded as described before or stalled at the pyrrolid‐2‐one intermediate **184**, even when an excess of PhSiH_3_ (4 equiv) was used [76]. Also, the polarity of starting materials could be easily reversed, for example, amino acids reacted with aldehydes under similar conditions to form pyrrolidines [77].

**SCHEME 47:**
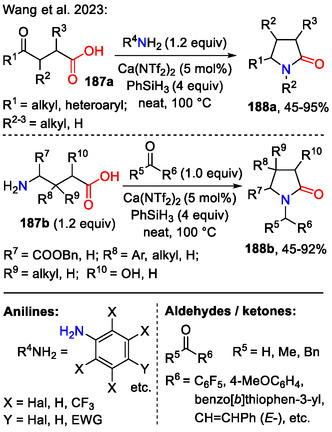
Synthesis of pyrrolidin‐2‐ones **188a‐b** by reductive amination.

Similarly, the reductive amination of biomass‐derived keto acids **187a** with amines in a Ca(NTf_2_)_2_ catalyzed process was used to directly access polyhalogenated pyrrolidine‐2‐ones **188a** (Scheme 47). Alternatively, pyrrolidine‐2‐ones **188b** were synthesized by reacting amino acids **187b** with carbonyl derivatives under the same conditions. Both approaches displayed chemospecificity and good substrate tolerance, offering a broad range of applications, which was demonstrated by late‐stage functionalization of drug molecules, such as *amlodipine*, *lenalidomide*, and *nabumetone* [78].

**SCHEME 48:**
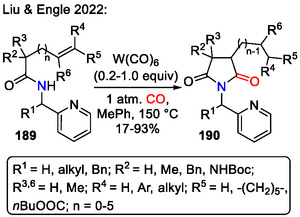
Tungsten redox catalysis for pyrrolidin‐2,5‐dione **190** synthesis via carbonylative functionalization.

**SCHEME 49:**
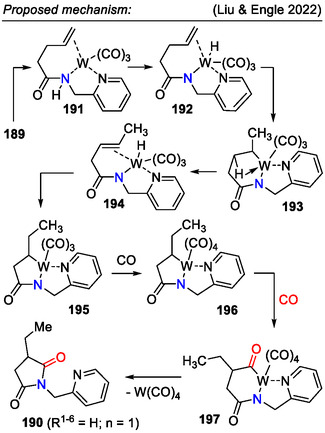
Mechanistic proposal for pyrrolidin‐2,5‐dione **190** synthesis via tungsten redox catalysis.

Pyrrolidin‐2,5‐diones **190** were accessed by isomerization and carbonylative functionalization of alkenes **189**, enabled by low‐valent tungsten redox catalysis (Scheme 48). In the presence of a W(CO)_6_ catalyst, the aminoalkene **189** C=C bond was isomerized to unactivated internal positions, resulting in regiospecific formation of pyrrolidine rings **190**, regardless of the substrate **189** C‐chain length (*n* = 0–5). The hydrocarbonylation step was performed under a CO atmosphere. The key mechanistic factors were six‐ to seven‐coordinate geometry changes, characteristic of the W(0)/W(II) redox cycle, and the conformationally flexible pyridin‐2‐ylmethyl directing group effect (Scheme 49) [79].

**SCHEME 50:**
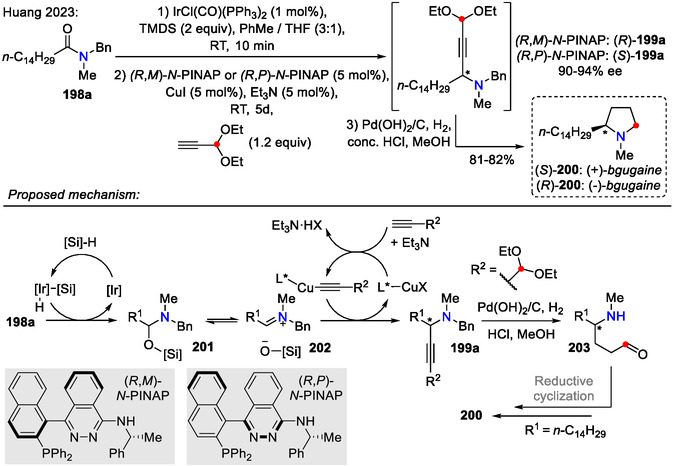
Enantioselective synthesis of α‐alkyl pyrrolidines **200**.

Huang and coworkers devised a one‐pot strategy for the enantioselective synthesis of α‐alkyl pyrrolidines **200** from amides **198a** and alkynes (Scheme 50). This method was employed for the total synthesis of chiral alkaloids, such as (‐)‐ and (+)‐*bgugaine* ((*R*)‐ and (*S*)‐**200**). Depending on the chirality of the *N*‐PINAP catalyst, the key intermediate **199a** could be obtained as either (*R*)‐ or (*S*)‐enantiomer, with enantioselectivity up to 94% ee. Then, reductive amination of an acetal group‐containing alkyne **199a** (*R* ^3^ = HC(OEt)_2_) provided either of the *bgugaine* isomers **200** [80].

The aliphatic amide **198a** enantioselective alkynylation step likely proceeded via the mechanism proposed by Huang and Wang, involving an Ir/Cu/chiral ligand (L*)‐catalyzed sequence (Scheme 50) [81]. Following tandem benzylic deprotection‐reductive cyclization step, using Pd(OH)_2_/C and H_2_, afforded pyrrolidine ring formation [80]. A mechanistically similar strategy was employed for the asymmetric synthesis of *anticaprant*—a κ‐opioid receptor antagonist—where *N*‐Boc‐*L*‐proline induced chirality instead of *N*‐PINAP catalysts [82].

**SCHEME 51:**
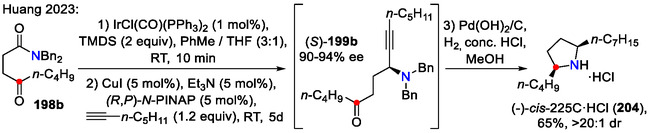
Enantioselective synthesis of α,α′‐dialkyl pyrrolidines **204**.

In the case of aliphatic chain‐containing alkynes (*R* ^3^ = alkyl), a γ‐carbonyl group present in the amide **198b** served as an electrophilic center for the internal nucleophile attack (Scheme 51). In this case, 2,5‐disubstituted pyrrolidine **204** was formed instead, as illustrated by the synthesis of the alkaloid (‐)‐*cis*‐225C (**204**) hydrochloride form [80].

## 5 Intramolecular Nucleophilic Substitution

Amino alcohols are common precursors for synthesizing the pyrrolidine scaffold via the *5‐exo‐tet* ring closing pathway. Conventionally, the alcohol is first converted into a good leaving group, such as a halide or sulfonate ester, whereas direct ring closure of amino alcohols is a less studied subject. Many substrates fail to tolerate the harsh conditions required (strong acids, heating) to displace the alcohol moiety with an incoming amine nucleophile [83], thus encouraging the development of milder activation methods.

**SCHEME 52:**
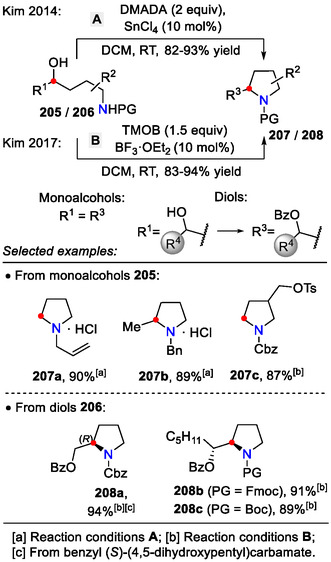
C─N bond formation in amino alcohols **205**/**206** by directly displacing the hydroxyl group.

**SCHEME 53:**
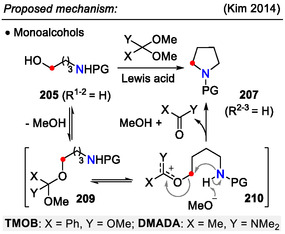
C─N bond formation in aminoalcohols.

**SCHEME 54:**
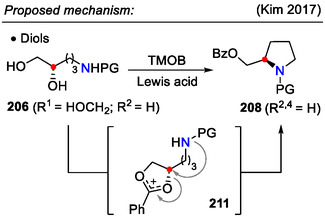
C─N bond formation in aminodiols **206**.

Direct alcohol displacement in amino alcohols **205** was achieved by combining trimethyl orthobenzoate (TMOB) with Lewis acidic BF_3_⋅OEt_2_ (Scheme 52). Under these conditions, the hydroxyl group was transformed into a leaving group, which was then displaced by an internal amide group to afford pyrrolidines **207** (Scheme 53). The reaction scope was extended from monoalcohols to vicinal diols **206** (*R* ^1^ = HOCHR^4^) as starting materials. Enantiomerically pure substrates (> 99% ee) afforded prolinol benzoates **208** (*R* ^3^ = BzOCH_2_) with complete inversion of configuration at the chiral center, suggesting a S_*N*_2‐like mechanism. The incorporation of the benzoate group into the product suggested a cyclic oxocarbenium intermediate **211**, formed on the diol moiety (Scheme 54) [84].

The same amino alcohol transformation (**205**→**207**) was achieved with *N*,*N*‐dimethylacetamide dimethyl acetal (DMADA) and SnCl_4_ (10 mol%) as the Lewis acid catalyst (Schemes 52 and 53). With comparable yields, HCl (1 equiv.) could also promote this reaction instead of the SnCl_4_ catalyst. A practical application for direct activation of alcohols using the DMADA / H^+^ system was demonstrated in the synthesis of natural product *crispine A* [83].

**SCHEME 55:**
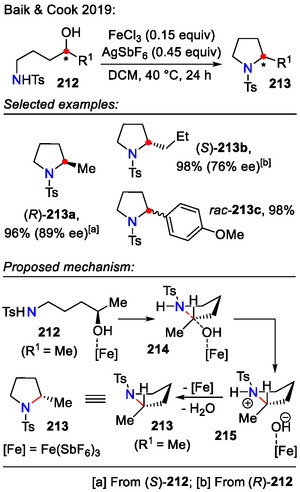
Iron‐catalyzed amino alcohol **212** heterocyclization into pyrrolidines **213**.

Alternatively, secondary alcohols **212** could be transformed in situ into leaving groups using iron(III)‐catalysis under non‐solvolytic conditions (Scheme 55). The iron‐catalized approach for C─N bond formation was advantageous because the only stoichiometric byproduct was water. Fe(SbF_6_)_3_ was proposed to be the active catalytic species since the best yields were observed when using three equivalents of AgSbF_6_ relative to Fe^3+^. It was also confirmed by DFT calculations that the energy barrier for C─N bond formation with iron catalysts decreased in the following order: FeCl_3_ (highest) > Fe(SbF_6_)Cl_2_ > Fe(SbF_6_)_2_Cl > Fe(SbF_6_)_3_ (lowest). The intramolecular displacement of chiral ROH‐[Fe] species with tethered sulfonamide nucleophiles in transition state **214** occurred with inversion of stereochemistry. This was ensured by applying the tight ion pairing concept, where the departing iron hydroxide blocked one face of the carbocation, favoring rapid attack of the sulfonamide from the opposite face, thus ensuring facial selectivity. This remained true for simple alkyl chains (*R* = Me, *n*Pr, *i*Bu); however, benzylic alcohols *R* = *p*‐MeOC_6_H_4_) gave racemic pyrrolidines **213c** instead, likely due to the formation of a stabilized benzylic carbocation intermediate [85].

As demonstrated by previous examples, C─N bond formation via intramolecular nucleophilic substitution offers the possibility of chiral pyrrolidine synthesis by inversion of stereochemistry. This requires chirality to be present at the reactive site of the starting material (**206**/**212**), which is not always easy to introduce. A convenient alternative for the stereoselective synthesis of 2‐substituted pyrrolidines involves the use of chiral sulfinimines **216** as starting materials [86, 87, 88, 89].

**SCHEME 56:**
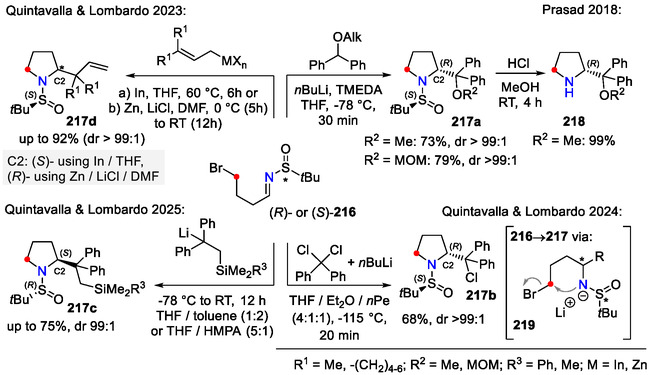
Diastereoselective synthesis of pyrrolidines **217a‐d**, containing chiral sulfinyl N‐auxilaries.

In one example by the Prasad group, deprotonated diphenylmethyl ethers were added to the chiral imine **216**, bearing an intrinsic leaving group (Scheme 56). The sulfinamide intermediate **219** further cyclized to afford pyrrolidine **217a**. Further treatment with HCl in MeOH afforded the enantiopure pyrrolidine organo‐catalyst **218** in a quantitative yield. Interestingly, the same conditions could also be used to construct heterocycles of smaller ring size, such as aziridines and azetidines. Piperidine ring formation was also possible, but under modified conditions (NaH in THF and heating for the cyclization step) [86].

Quintavalla and Lombardo switched to the carbenoid (chlorodiphenylmethyl)lithium species to diastereoselectively obtain in the side chain chlorinated pyrrolidine **217b** (Scheme 56). In this case, the reactive organolithium species was generated via chlorine‐lithium exchange. The obtained chlorinated pyrrolidines **217b** were further converted to ether‐type products **217a** by S_*N*_1 reaction with a variety of alcohols [87].

A similar approach was used for the synthesis of silyl‐substituted pyrrolidines **217c** (Scheme 56). Enantiopure (*R*)‐sulfinimine **216** reacted with the silyl‐substituted diphenylethyllithium reagent and underwent cyclization to afford (2*S*)‐stereocenter‐containing pyrrolidine **217c**. Before addition, the organolithium reagent was prepared in situ by silyllithium reagent R^3^Me_2_SiLi addition to 1,1‐diphenylethylene. The (*S*)‐specific stereochemistry for the newly formed stereocenter (C2) was attributed to an open antiperiplanar transition state for the nucleophilic addition to the imine step. This method was applied for the synthesis of Hayashi−Jørgensen organocatalyst methylene isosteres [88].

Diastereoselective allylation of chiral sulfinyl imines **216**, followed by intramolecular cyclization, afforded the synthetic precursors for *α*‐disubstituted β‐homoprolines **217d** (Scheme 56). The absolute configuration of the newly formed stereocenter at the C2 position was modified depending on the allylation protocol: (a) In(0) in THF; or b) Zn(0), LiCl in DMF. Alternatively, the C2 position stereochemistry could also be modulated by using a *tert*‐butanesufinimide chiral auxiliary with reverse configuration [89].

**SCHEME 57:**
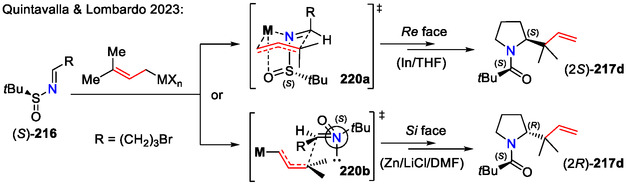
Transition states for allyl indium and allyl zinc addition to chiral sulfinimine **216**.

The stereochemistry for the C2 position in pyrrolidine **217d** could be explained by two distinct transition states (Scheme 57). The in situ generated allyl indium reagent reacted with the chiral imine **216** via a closed six‐membered transition state **220a**, whereas the allyl zinc reagent in strongly coordinating solvents (DMF) and in the presence of LiCl favored an open antiperiplanar transition state **220b** [89].

## 6 C(sp^3^)‐H Activation in Alkylamines

**SCHEME 58:**
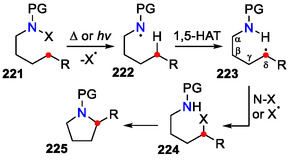
General concept for the HLF reaction.

The concept of remote C_δ_(sp^3^)‐H activation at otherwise unfunctionalized positions in *N*‐haloamines **221**, resulting in heterocyclic five‐membered ring **225** formation, namely, the Hofmann‐Löffler‐Freytag (HLF) reaction, is known for over a century (Scheme 58) [90, 91]. The original harsh conditions proposed by Löffler and Freytag [92, 93] limited wider application; therefore, several modifications to this reaction have been studied since.

Noteworthy examples include the in situ formation of *N*‐iodinated sulfonamides, by combining iodine(III) reagents with catalytic amounts of I_2_ under visible light irradiation [94]. However, using molecular iodine often led to undesired decomposition; therefore, the Nagib group suggested generating I_2_ in situ by oxidation of I^‐^ salts [95]. Copper catalysts could be used instead of halide sources, as was demonstrated by Yang and coworkers [96]. These and some other examples, involving iodine(III) reagents, were discussed in detail in the recently published review by Wang et al. [97].

**SCHEME 59:**
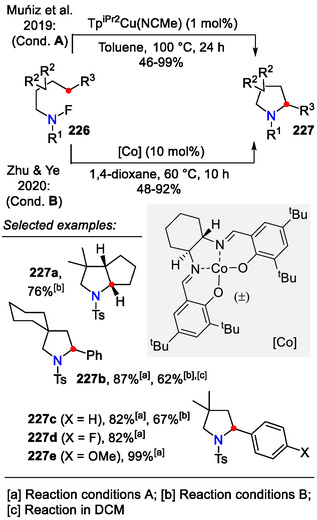
HLF‐type C_δ_(sp^3^)‐H functionalization in *N*‐fluorinated sulfonamides **226**.

Surprisingly, *N*‐fluorinated sulfonamides have not found much application in the field of HLF‐type C_δ_(sp^3^)‐H functionalizations. In contrast to its heavier halogen analogs, N─F bonds have a lower tendency for homolytic cleavage, required for the HLF‐type reaction pathway. Using copper(I) catalysts, which are known to promote single‐electron transfer (SET), Muñiz et al. achieved N─F bond cleavage and subsequent intramolecular amination, resulting in pyrrolidines **227** (Scheme 59; conditions A) [98]. Cobalt(II) catalysts also facilitated this transformation, as it was demonstrated by Zhu and Ye (conditions B) [99].

**SCHEME 60:**
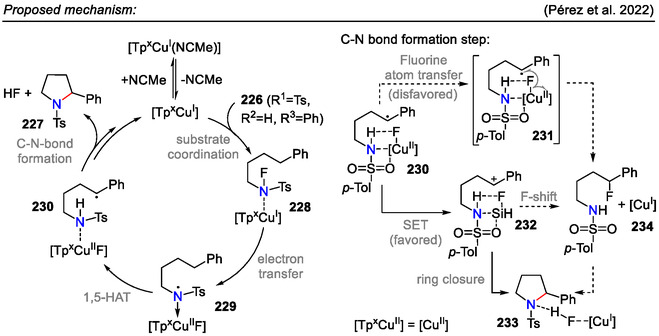
Catalytic cycle and C─N bond formation step for the HLF‐type reaction in *N*‐fluorinated sulfonamides **226**.

Further mechanistic studies elucidated that the heterocyclization step proceeded through intermediate **230**, which was further oxidized to a cationic species **232** via a SET mechanism, followed by ring closure and HF formation (Scheme 60). It was also demonstrated that the fluoroalkane **234** was not an intermediate in the catalytic formation of pyrrolidines from the N─F reactants [100].

Furthermore, using *N*‐fluorinated substrates **226** was preferential to analogical *N*‐chlorinated substrates due to more favorable reaction pathways, which was reflected in product **227** yields. For instance, *N*‐Cl‐sulfonamide (*R* ^1^ = Ts, *R* ^2^ = Me, *R* ^3^ = Ph), when subjected to identical reaction conditions (1 mol% Tp^iPr2^Cu(NCMe), toluene, 100 °C, 24 h, afforded pyrrolidine **227** in 83% yield (NMR); meanwhile, its N‐F fluorinated analog **226** afforded pyrrolidine **227** with 99% yield by NMR [100].

**SCHEME 61:**
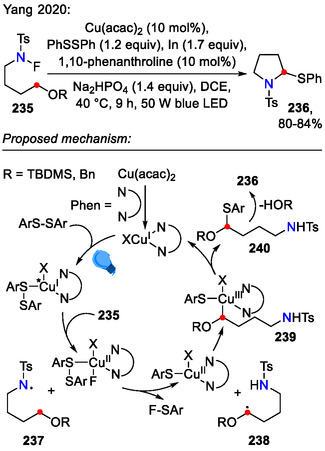
Synthesis of 2‐PhS‐substituted pyrrolidines **236**.

A modified catalytic system for the HLF‐type reaction was proposed by Yang and coworkers, who reacted *N*‐fluorinated aliphatic amines **235** with phenyl disulfide in the presence of Cu(acac)_2_, 1,10‐phenanthroline, Na_2_HPO_4_ and indium powder under blue LED light (Scheme 61). This transformation afforded 2‐phenylthio‐substituted pyrrolidines **236** via site‐selective C(sp^3^)–S bond formation. For the reaction to yield cyclization products **236**, the substrate needed to contain the OTBDMS or OBn group at the C(4) position; otherwise, acyclic sulfides were obtained instead. Control experiments demonstrated that the reaction could proceed in the absence of light, but with a significant decrease in yield [101].

**SCHEME 62:**
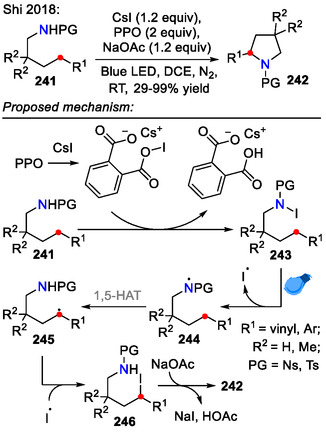
HLF‐type reaction, including in situ generation of *N*‐iodinated amide **243**.

While the *N*‐fluorinated amide starting materials could be sufficiently easily prepared from the respective sulfonamides by deprotonation with NaH (6 equiv) and subsequent treatment with NFSI (3 equiv) [100], it is much more convenient to generate the *N*‐halogenated amides in situ*.* Such was the case in the photochemical approach devised by the Shi group, where phthaloyl peroxide (PPO) was combined with iodide salts as a halogenating system to perform the HLF‐type reaction on amides **241** (Scheme 62). Both electron‐rich and electron‐poor benzylic systems (*R* ^1^ = Ar) were applicable for this reaction, with one example of allylic C─H functionalization (*R* ^1^ = vinyl) [102].

**SCHEME 63:**
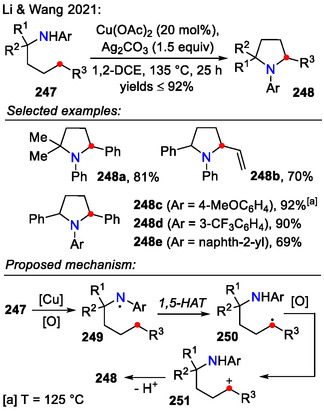
Copper‐catalyzed halogen‐free approach towards pyrrolidine **248** synthesis via C─H activation.

Li and Wang proposed a halogen‐free approach toward direct C_δ_(sp^3^)‐H amination in arylamines **247**, containing either a benzylic or an allylic site (Scheme 63). Cu(OAc)_2_ was used to generate an *N*‐centered radical **249**, which subsequently rearranged to the stabilized benzylic/allylic radical **250** via a 1,5‐HAT process. The in situ‐generated radical **250** was then oxidized to carbenium ion **251**, which then underwent intramolecular amination. In addition to five‐membered ring formation, as commonly observed in HLF reaction pathways, the ring size could be altered to obtain four‐ and six‐membered azacycles during the 1,n‐HAT process [91].

**SCHEME 64:**
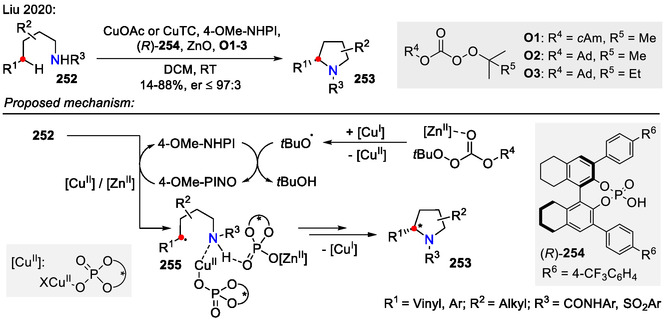
Asymmetric pyrrolidine **253** synthesis by C(sp^3^)‐H activation.

An asymmetric version of the copper‐catalyzed Hofmann‐Löffler‐Freytag‐type reaction was reported by Liu and coworkers (Scheme 64). By using a redox Cu(I) / chiral phosphoric acid catalytic system in combination with 4‐Methoxy‐NHPI (NHPI = *N*‐hydroxyphthalimide) as HAT mediator precursor, enantioselective intramolecular C(sp^3^)‐H amination of allylic and benzylic positions was achieved, yielding 2*S*‐pyrrolidines **253**. In the proposed reaction mechanism, the Cu(I) catalyst reacted with the Zn‐complex‐activated peroxide to generate the alkoxy radical (*t*BuO⋅) and the chiral Cu(II) phosphate complex. The formed alkoxy radical reacted further with 4‐MeO‐NHPI to provide the 4‐methoxy‐PINO (PINO = phthalimide *N*‐oxyl) radical. In the presence of the chiral Cu(II) and Zn(II) phosphate complexes, 4‐MeO‐PINO selectively abstracted the hydrogen atom from allylic/benzylic position of the substrate **252** to produce the stabilized radical **255**. The latter underwent oxidation by Cu(II), followed by ring closure to afford chiral pyrrolidine **253** [103].

**SCHEME 65:**
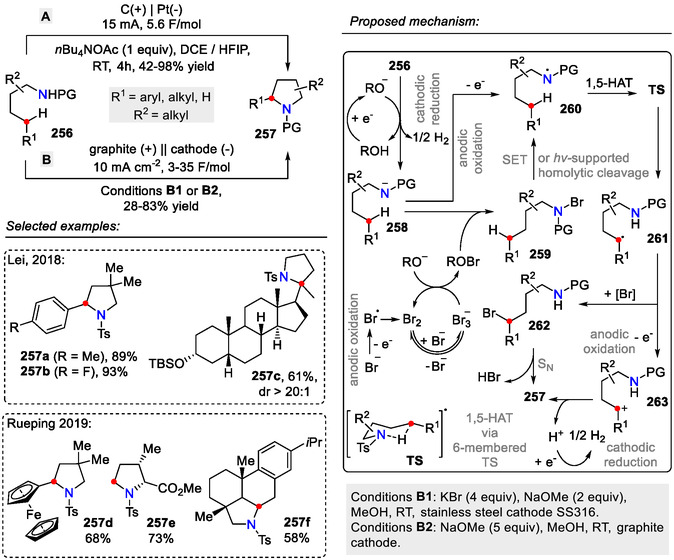
Electrochemical C_δ_(sp^3^)‐H amination for the synthesis of pyrrolidines **257**.

Some electrochemical approaches have been demonstrated as well, obviating the need for metal catalysts, halogenated reagents, and stoichiometric additional oxidants. Lei and coworkers employed a C(+)|Pt(‐) system under constant‐current electrolysis conditions (15 mA) for oxidative intramolecular C(sp^3^)‐H bond amination of sulfonamides **256**, resulting in pyrrolidines **257** (Scheme 65; conditions A) [104]. A similar system for electrochemical synthesis of pyrrolidines **257** was devised by Muñiz and coworkers, where Bu_4_NBF_4_ was used as the supporting electrolyte. Gratifyingly, they managed to decrease the electric current rate to I = 2.5 mA (2.2 F), with only a slight increase in reaction time (4.7 h) [105].

The Rueping group demonstrated that the electrochemical dehydrogenative C(sp^3^)‐H amination (**256**→**257**) could be performed under both bromide‐mediated and halogen‐free conditions (Scheme 65; conditions B). This approach was applicable for batch and continuous flow processes. Good functional group tolerance and regioselectivity were observed for this method, allowing the synthesis of pyrrolidines **257**, containing more exotic functional groups, for example, 2‐ferrocenylpyrrolidine **257d**. Large‐scale synthesis was also possible—the anodic transformation of Ts‐*iso*‐Leu‐OMe was performed on a 50 g scale, obtaining *N‐*tosyl‐3‐methyl‐prolinecarboxylate **257e** with 73% yield [106].

Some key differences could be observed in the mechanism for pyrrolidine **257** formation via the bromine‐mediated and halogen‐free electrochemical processes (Scheme 65). In the bromine‐mediated reaction, the intermediate *N*‐radical **260** was obtained by homolysis of the *N*‐Br species **259**, generated in situ by reacting the amide anion **258** with methyl hypobromite (ROBr), where the latter was also electrochemically generated in the reaction mixture. After the 1,5‐HAT event, the C‐centered radical **261** was quenched by the MeOBr or *N*‐Br species **259** (although a simple anodic oxidation to the carbocation was also plausible), and a nucleophilic substitution event afforded the pyrrolidine scaffold **257**. In the halogen‐free approach, the *N*‐radical **260** generation occurred by anodic oxidation of the amide anion **258**. In the same manner, the C‐centered radical **261** was oxidized to carbocation **263**. Subsequent ring closure by nucleophilic attack of the internal amide afforded pyrrolidine **257** [106].

Combining electricity and light in a synergistic catalysis approach is an emerging paradigm in heterocycle synthesis [107]. This approach obviates the need for chemical oxidants and, in some cases, enhances reaction selectivity [108].

**SCHEME 66:**
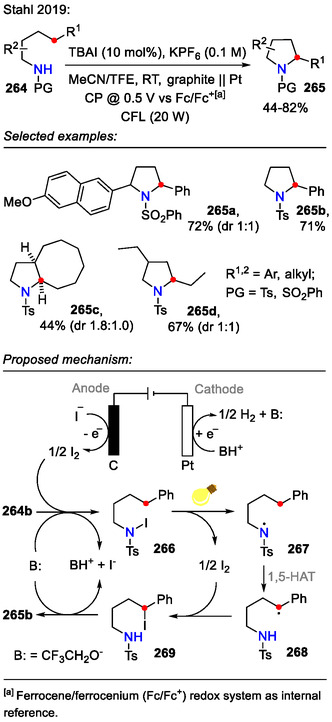
Electrochemical amination of C(sp^3^)−H bonds incorporating visible light.

In an illustrative example by the Stahl research group, a photo/electrochemical iodide‐mediated strategy was developed for the synthesis of pyrrolidines **265** (Scheme 66). The reaction was performed under constant potential (CP) conditions using KPF_6_ (0.1 M) as the supporting electrolyte. Tetra‐*n*‐butylammonium iodide (TBAI, 10 mol%) as an electrochemical mediator allowed the electrochemical process to proceed at low electrode potentials, ensuring reaction selectivity for substrates **264** bearing electron‐rich substituents, which were otherwise prone to unwanted side reactions. This was highlighted by the synthesis of pyrrolidine **265a**, bearing the electron‐rich methoxynaphthyl substituent (72% yield). For comparison, only negligible C(sp^3^)‐H activation was observed (**265a**, <10%) [109] when using the earlier discussed HLF‐reaction conditions provided by Muñiz [105] and Lei [104].

The electrochemical part of the reaction mechanism is based on anodic generation of I_2_ from an iodide (I^‐^). Hydrogen evolution occurred on the cathode with additional generation of a Brønsted base (CF_3_CH_2_O^‐^), which is required to promote substrate **264** N‐H iodination. In bulk solution, photolytic homolysis of the N─I bond enabled a C(sp^3^)‐H functionalization sequence, involving sequential *N*‐centered radical **267** formation, a 1,5‐HAT event to generate an alkyl radical **268** and alkyl iodide **269** generation. The sequence was finalized by a Brønsted base‐promoted cyclization to form the pyrrolidine scaffold **265** [109].

**SCHEME 67:**
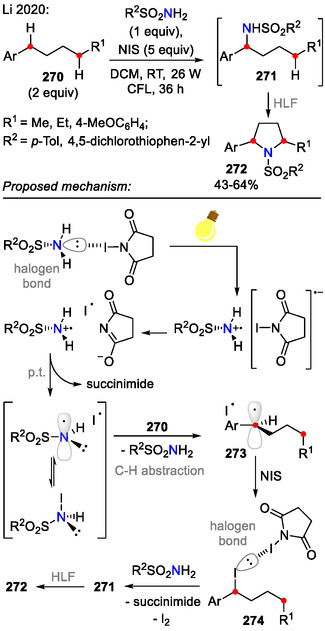
Pyrrolidine **272** synthesis from hydrocarbons **264** by two sequential C(sp^3^)‐H aminations.

One of the main limitations for the intramolecular C─N bond formation strategy remains complex starting material synthesis, requiring the incorporation of an electrophilic center and the nucleophilic amine functionality for the heterocyclization to occur. Li and coworkers addressed this problem by devising a strategy for two consecutive C(sp^3^)‐H aminations in simple hydrocarbons **270**, which are easily accessible starting materials (Scheme 67). This was achieved through a halogen bonding charge–transfer complex, which initiated visible light absorption, inducing *N*‐radical formation in sulfonamides and a subsequent HAT process. Two consecutive C(sp^3^)‐H aminations took place, forming pyrrolidines **272**. The reaction intermediates*—N*‐benzyl sulfonamides **271**—could be isolated with yields up to 98% if the reaction was quenched after 16 h and the used *N*‐succinimide (NIS) amount did not exceed 3 equiv. This approach was used to derivatize the sulfonamide moiety‐containing anti‐inflammatory drug *Celebrex*, introducing a pyrrolidine ring in its molecule [110].

**SCHEME 68:**
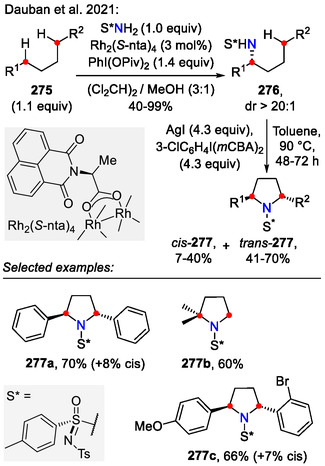
Diastereoselective strategy for pyrrolidine **271** synthesis by two sequential C(sp^3^)‐H functionalizations.

Lastly, Dauban and coworkers demonstrated a diastereoselective strategy for pyrrolidine **277** synthesis by two sequential C(sp^3^)‐H functionalizations of simple hydrocarbons **275** (Scheme 68). A regio‐ and stereoselective nitrene C─H insertion (**275→276**) was performed using a chiral rhodium catalyst and iodine(III) reagent. To achieve regioselective amination of the benzylic site in non‐symmetrical hydrocarbons **275**, the aromatic groups had to be differentiated by the introduction of electron‐donating or withdrawing substituents, for example, the first benzylic amination occurred preferentially next to the more electron‐rich aryl group. While dibenzylic substrates (*R* ^1,2^ = aryl) provided higher pyrrolidine **277** yields (up to 70%), aliphatic substrates also underwent this transformation, as demonstrated by example **277b** [111].

**SCHEME 69:**
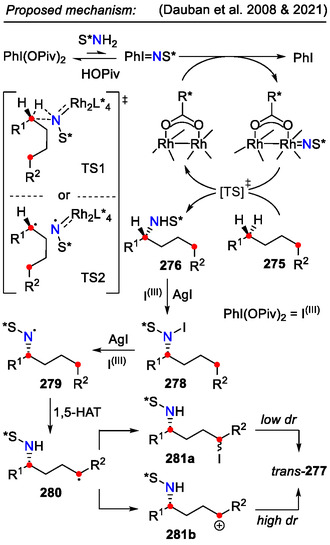
Mechanistic proposal for diastereoselective pyrrolidine **277** synthesis by two sequential C(sp^3^)‐H functionalizations.

The mechanism for stereoselective nitrene C─H insertion involved three main mechanistic steps: 1) iminoiodane S*N = IPh generation from PhI(OPiv)_2_ and chiral auxiliary‐containing amide S*NH_2_; 2) rhodium(II) complex oxidation by the iminoiodane; and C─H insertion into the formed rhodium‐nitrene intermediate S*N = [Rh] (Scheme 69). Two distinct transition states were proposed for the latter transformation, featuring 1) asynchronous concerted C─H insertion of the metallocarbene ([TS1]^‡^); or 2) H‐abstraction/radical rebound mechanism (TS2]^‡^) [112]. Control experiments on the *N*‐iodoamide **278** *N*‐chlorinated analog suggested a synergistic effect between AgI and the iodine(III) reagent in advancing the reaction onward. Subsequent HLF‐type cyclization, involving 1,5‐hydrogen atom transfer (HAT) from the *N*‐centered radical **279**, led to the diastereoselective formation of pyrrolidines **277**. The involvement of a cationic intermediate **281b** for the cyclization step was indicated by the obtained diastereomeric ratios (dr ≤ 8:1 *trans*/*cis*). The alternative S_*N*_2‐type cyclization route via intermediate **281a** was excluded, as radical trapping far from the stereogenic center would occur with poor stereocontrol [111].

Afterwards, the sulfonimidamide *N*‐protecting group, which served as a chiral auxiliary for the heterocyclization step (**276**→**277**), could be conveniently removed using Mg/MeOH under sonification, affording *trans*−2,5‐disubstituted pyrrolidines with further N−H functionalization possibilities [111].

## 7 Summary and Outlook

In this review, we summarize methodologies for intramolecular C─N bond formation in systems that combine a nucleophilic nitrogen center with an electrophilic carbon species, leading to the synthesis of pyrrolidine derivatives. The survey covers literature published between 2013 and 2025, including examples for 1) intramolecular amination of double and triple bonds; 2) tandem annulation strategies, featuring intramolecular C─N bond formation as key step; 3) *N*‐nucleophile additions to C=O groups; 4) intramolecular nucleophilic substitution and 5) C(sp^3^)‐H activation strategies, leading to pyrrolidines.

During the covered timeframe, significant progress has been made toward addressing key challenges associated with intramolecular C─N bond formation, including the structural complexity of starting materials, the need for side chain functionalization, as well as regio‐ and stereoselectivity control. Considerable effort has been directed toward incorporating principles of sustainable synthesis—catalytic reaction design, reduced reagent toxicity, and streamlined synthetic sequences to minimize the overall number of required synthetic steps, with electrochemical approaches providing particularly notable examples.

Of special interest is the emerging potential of regio‐ and stereooselective C(sp^3^)–H bond functionalization, which enables access to pyrrolidines from more readily available substrates, including alkylamines and hydrocarbons. Rare but important contributions to asymmetric pyrrolidine synthesis via C(sp^3^)–H amination are also highlighted.

## Funding

This study was supported by Latvijas Zinātnes Padome (LZP‐2023/1‐0576); Central Finance and Contracting Agency of Republic of Latvia (5.2.1.1.i.0/2/24/I/CFLA/003, ID 1134).

## Conflicts of Interest

The authors declare no conflicts of interest.
